# Characterising metabolomic signatures of lipid-modifying therapies through drug target mendelian randomisation

**DOI:** 10.1371/journal.pbio.3001547

**Published:** 2022-02-25

**Authors:** Tom G. Richardson, Genevieve M. Leyden, Qin Wang, Joshua A. Bell, Benjamin Elsworth, George Davey Smith, Michael V. Holmes

**Affiliations:** 1 MRC Integrative Epidemiology Unit (IEU), Population Health Sciences, Bristol Medical School, University of Bristol, Oakfield House, Oakfield Grove, Bristol, United Kingdom; 2 Novo Nordisk Research Centre, Headington, Oxford, United Kingdom; 3 Bristol Medical School: Translational Health Sciences, Dorothy Hodgkin Building, University of Bristol, Bristol, United Kingdom; 4 MRC Population Health Research Unit (PHRU), Clinical Trial Service Unit & Epidemiological Studies Unit, Nuffield Department of Population Health, University of Oxford, Oxford, United Kingdom; Duke University, UNITED STATES

## Abstract

Large-scale molecular profiling and genotyping provide a unique opportunity to systematically compare the genetically predicted effects of therapeutic targets on the human metabolome. We firstly constructed genetic risk scores for 8 drug targets on the basis that they primarily modify low-density lipoprotein (LDL) cholesterol (HMGCR, PCKS9, and NPC1L1*)*, high-density lipoprotein (HDL) cholesterol (CETP), or triglycerides (APOC3, ANGPTL3, ANGPTL4, and LPL). Conducting mendelian randomisation (MR) provided strong evidence of an effect of drug-based genetic scores on coronary artery disease (CAD) risk with the exception of ANGPTL3. We then systematically estimated the effects of each score on 249 metabolic traits derived using blood samples from an unprecedented sample size of up to 115,082 UK Biobank participants. Genetically predicted effects were generally consistent among drug targets, which were intended to modify the same lipoprotein lipid trait. For example, the linear fit for the MR estimates on all 249 metabolic traits for genetically predicted inhibition of LDL cholesterol lowering targets HMGCR and PCSK9 was r^2^ = 0.91. In contrast, comparisons between drug classes that were designed to modify discrete lipoprotein traits typically had very different effects on metabolic signatures (for instance, HMGCR versus each of the 4 triglyceride targets all had r^2^ < 0.02). Furthermore, we highlight this discrepancy for specific metabolic traits, for example, finding that LDL cholesterol lowering therapies typically had a weak effect on glycoprotein acetyls, a marker of inflammation, whereas triglyceride modifying therapies assessed provided evidence of a strong effect on lowering levels of this inflammatory biomarker. Our findings indicate that genetically predicted perturbations of these drug targets on the blood metabolome can drastically differ, despite largely consistent effects on risk of CAD, with potential implications for biomarkers in clinical development and measuring treatment response.

## Introduction

Cardiovascular disease (CVD), including coronary artery disease (CAD) and ischaemic stroke, is the leading cause of death worldwide [1]. Circulating lipoprotein lipid concentrations are of central importance to the aetiology of CAD [2,3]. For example, clinical trials [4] and studies of human genetics [5–7] converge to support a causal role of apolipoprotein B (apoB) and low-density lipoprotein (LDL) cholesterol concentrations in the initial development and subsequent progression of CAD.

Pharmacological therapies that target the metabolism of blood lipids are routinely used for the prevention and treatment of CVD and are among the most widely prescribed medicines in the world [8]. Interestingly, drug targets that modify concentrations of LDL cholesterol (for instance, statins, acting on HMG-CoA reductase [HMGCR]) and those designed to modify high-density lipoprotein (HDL) cholesterol (for instance, cholesteryl ester transfer protein [CETP] inhibitors) and triglycerides (for instance, angiopoietin-like protein 3 [ANGPLT3] inhibitors) act on discrete pathways involved in lipid metabolism. Therefore, while each of these drug classes has proven [9–11] or emerging [12–15] efficacy for CVD risk reduction, their effects on the blood lipidome and metabolome are likely to vary considerably. This has implications on understanding which biomarkers to measure (for instance, during clinical development in randomised controlled trials) and on gauging markers of treatment response [16].

**Fig 1:**
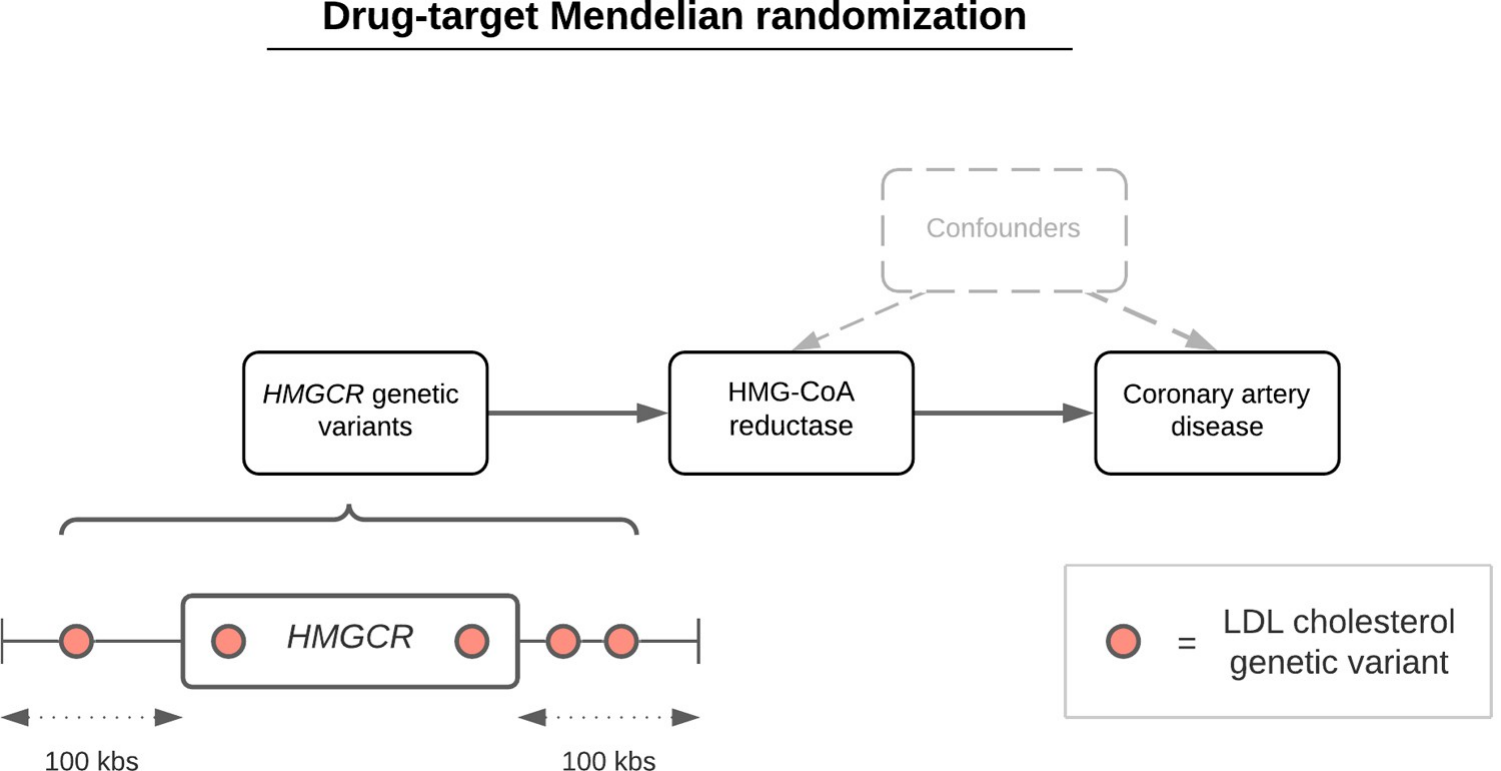
A schematic representation of the drug-target MR approach undertaken in this study using, for instance, HMGCR variants to proxy for HMG-CoA reductase inhibition (the mechanism of action of statin therapy) to estimate its genetically predicted effect on CAD. Genetic variants robustly associated with a lipoprotein lipid trait (for instance, LDL cholesterol) based on *P* < 1 × 10^−6^ within 100 kbs of encoding genes were identified as genetic proxies for perturbing therapeutic targets. A sensitivity analysis restricted to 50 kbs on either side of encoding genes was also undertaken in this study. CAD, coronary artery disease; kbs, kilobases; LDL, low-density lipoprotein; MR, mendelian randomisation.

In this study, we sought to estimate the effects of lipid-modifying therapeutic targets on the blood metabolome to better characterise their impact on biomarkers related to CVD risk reduction. We constructed genetic instruments for drug targets that are either currently licenced or under development and grouped them according to their primary lipid of pharmacological focus: LDL cholesterol, HDL cholesterol, or triglycerides. We then compared the genetically predicted effects of therapeutic targets on CAD risk, before evaluating their effects on circulating lipoprotein lipid concentrations newly measured at large scale in the UK Biobank (UKB) study through the application of drug-target mendelian randomisation (MR) [17–19] (**Fig 1**).

## Results

### Genetic instrumentation of lipid-modifying drug targets to estimate their therapeutic effects on coronary artery disease and type 2 diabetes risk

We conducted genome-wide association studies (GWAS) on measures of LDL cholesterol (*n =* 328,111), HDL cholesterol (*n* = 300,528), and triglycerides (*n* = 328,498) in the UKB using biochemistry measures of these traits. Sample sizes were determined based on standard exclusion criteria (see Materials and methods), as well as excluding participants with measures of metabolic traits derived from a newly available nuclear magnetic resonance (NMR) platform in UKB. This was to avoid overlapping samples in our planned MR analyses of metabolic traits, which has been reported to potentially lead to overfitting in estimates [20]. Instrumental variables for 8 drug targets were identified using results of these GWAS for planned MR analyses. These were *PCSK9*, *HMGCR*, and *NPC1L1* [4] (using LDL cholesterol results), *CETP* (using HDL cholesterol results), and *APOC3*, *ANGPTL3*, *ANGPTL4*, and *LPL* [15,21] (using triglyceride results). In total, we identified 137 instruments based on *P* < 1 × 10^−6^, r^2^ < 0.1, and a window size of 100 kbs either side of the 8 genetic loci responsible for encoding the lipid-modifying targets evaluated in this study (**S1 Table**).

**Fig 2:**
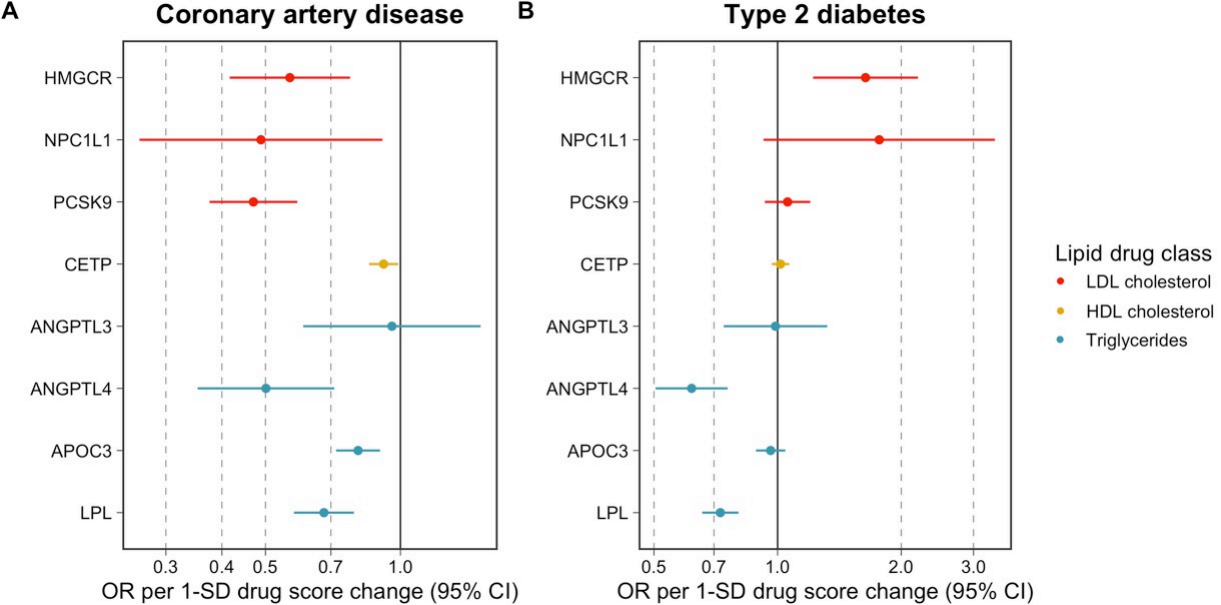
A forest plot visualising the genetically predicted effects of lipid-modifying drug targets on risk of CAD and T2D using MR. Estimates are colour coded based on the lipoprotein lipid trait estimates used to derive genetic scores. Each genetic score was oriented to mimic the putative effects of drug targets on lipoprotein traits, meaning that effect estimates correspond to relative odds of disease per 1 SD change in either lower LDL cholesterol, higher HDL cholesterol, or lower triglyceride levels via each specific drug target. Note that in the case of CETP, we are not ascribing causal effects to HDL cholesterol—rather, we are orientating CAD/T2D effect estimates corresponding to a genetically predicted increase in HDL cholesterol arising from pharmacological inhibition of CETP. The data underlying this figure can be found in **S2 Table**. CAD, coronary artery disease; CETP, cholesteryl ester transfer protein; HDL, high-density lipoprotein; LDL, low-density lipoprotein; MR, mendelian randomisation; SD, standard deviation; T2D, type 2 diabetes.

Two-sample MR analyses were undertaken using the inverse variance weighted (IVW) method while accounting for the correlation between instruments [22,23] (**Fig 1**). Using results on 60,801 CAD cases and 123,504 control from the CARDIoGRAMplusC4D consortium, we found strong evidence of a genetically predicted effect for each therapeutic target on CAD risk (based on false discovery rate (FDR) < 5%) with the exception of *ANGPTL3* (**S2 Table** and **Fig 2**), in keeping with prior findings [5,24–28]. Likewise, analyses on type 2 diabetes (T2D) risk using results from a GWAS of 74,124 cases and 824,006 controls from the DIAMANTE consortium supported previous findings (**S2 Table**). For instance, this included a genetically predicted adverse effect for the *HMGCR* score with T2D risk (OR = 1.64, 95% CI = 1.22 to 2.20, *P* = 0.001), whereas a protective effect was found for the *LPL* score (OR = 0.73, 95% CI = 0.66 to 0.80, *P* = 6.05 × 10^−10^). There was additionally strong evidence of a genetically predicted effect on T2D risk for the *ANGPTL4* score (OR = 0.62, 95% CI = 0.50 to 0.76, *P* = 2.65 × 10^−6^). F-statistics did not indicate that drug target scores were prone to weak instrument bias (F = 58.3 to 297.1) (**S2 Table**). Genetically predicted effects on LDL cholesterol, HDL cholesterol, and triglycerides based on UKB biochemistry measures in the participants with NMR metabolites data (i.e., nonoverlapping with the partitioned sample, which instruments were selected in) can be found in **S3 Table**.

### Systematic evaluation of genetically predicted therapeutic target effects on metabolic traits

Next, we applied our GWAS pipeline to all 249 metabolic traits measured by targeted high-throughput NMR metabolomics from Nightingale Health (biomarker quantification version 2020) in the separate subset of UKB participants with these measures. Sample sizes after QC ranged between *n =* 110,051 to *n* = 115,082 (**S4 Table**). In total, there were 2,814 genetic variants robustly associated with at least one measure (based on the conventional GWAS cutoff *P* < 5 × 10^−8^) across 721 independent genetic loci (**S5 Table**). All of the 249 metabolic traits were represented among these findings (i.e., each trait quantified by the NMR platform had at least one SNP association at GWAS levels of significance) with the majority having dozens of independent variants associated with their levels (median: 74 variants, interquartile range: 27 variants) (**S6 Table**).

Systematically estimating genetically predicted effects of each lipid-modifying target in turn on each of the 249 metabolic traits using MR identified a total of 1,588 effects robust to FDR < 5% corrections (**S7–S14 Tables**). Investigating the robustness of our results to a more stringent instrument selection criteria (i.e., 50 kbs either side of encoding genes as compared to 100 kbs in our main analyses) provided strong evidence of homogeneity between genetically predicted drug-target effects in the original analysis (**S1–S8 Figs**).

**Fig 3:**
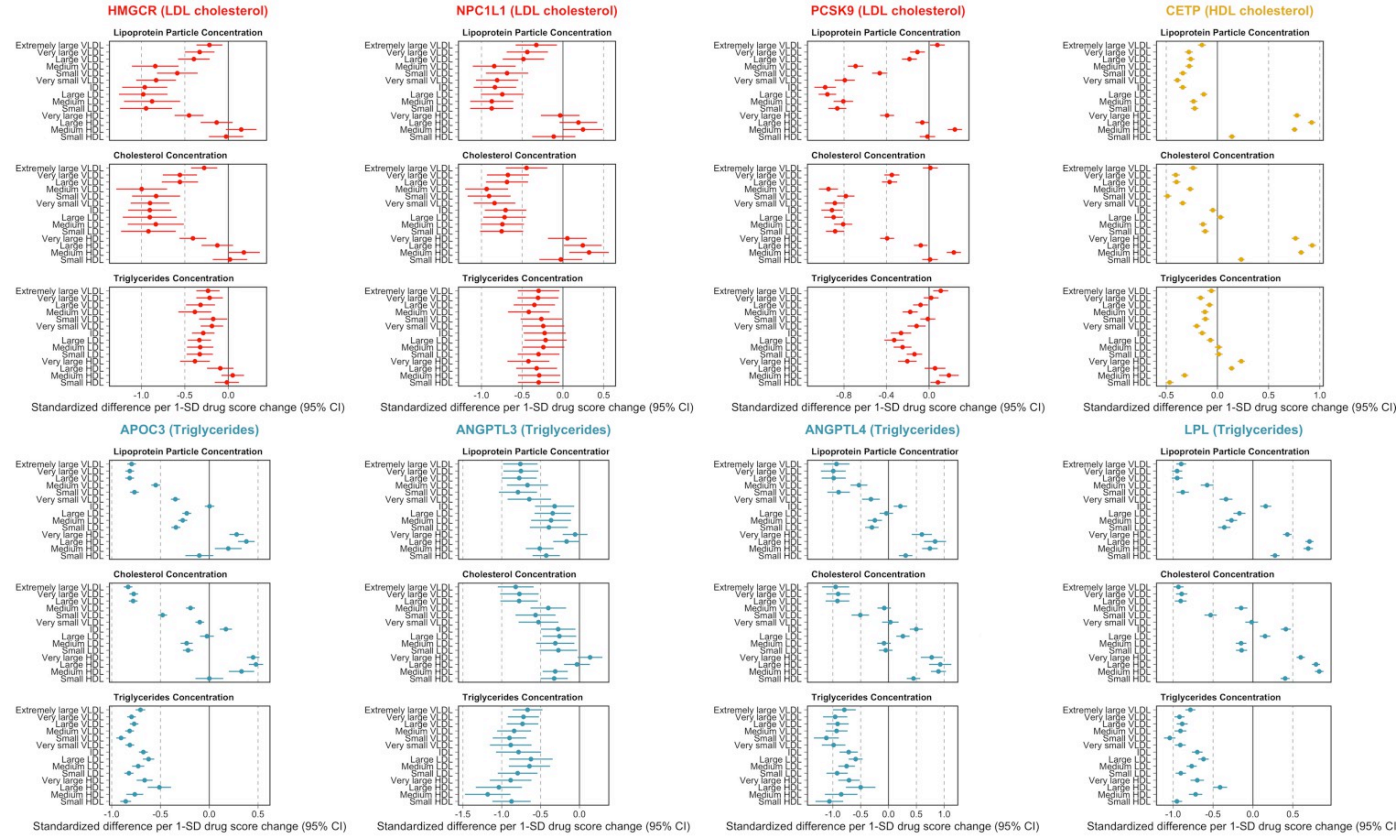
Forest plots illustrating the genetically predicted effects of lipid-modifying drug targets on measures of circulating metabolite concentrations using NMR in the UKB study. Effect estimates are based on an SD change in the genetically predicted drug target scores oriented to reflect therapeutic intervention (i.e., lower LDL cholesterol, lower triglycerides, and higher HDL cholesterol). Scores were derived using genetic variants robustly associated which lipoprotein lipid traits (as indicated in each target’s legend) at each encoding gene’s region. The data underlying this figure can be found in **S7–S14 Tables**. CETP, cholesteryl ester transfer protein; HDL, high-density lipoprotein; IDL, intermediate density lipoprotein; LDL, low-density lipoprotein; NMR, nuclear magnetic resonance; SD, standard deviation; UKB, UK Biobank; VLDL, very low-density lipoprotein.

A subset of these estimates related to lipoprotein particle, cholesterol, and triglyceride concentrations across the 8 drug targets have been highlighted in **Fig 3**. Broadly, the LDL cholesterol modifying targets (*HMGCR*, *PCSK9*, and *NPC1L1*) provided evidence of genetically predicted effects on lower levels of very low-density lipoprotein (VLDL), intermediate density lipoprotein (IDL), and LDL-related particle concentrations, but typically weak evidence on HDL-related markers (with the exception of very large HDL particles, for which genetic instruments for both *HMGCR* and *PCSK9* showed strong evidence of lowering). For example, the strongest evidence identified using the *PCSK9* score was on large LDL particle concentrations (Beta = 0.96 SD reduced per 1-SD lowering in LDL cholesterol, 95% CI = 0.87 to 1.04, *P* = 3 × 10^-113^). The concentration of cholesterol within lipoprotein particle subclasses tended to mimic the associations identified for lipoprotein particle concentration. In contrast, generally weaker effects of genetic instruments for *HMGCR*, *PCSK9*, and *NPC1L1* were identified for triglyceride concentrations within the same lipoprotein particle subclasses.

Orientated to a lowering of CAD risk, the HDL cholesterol modifying target *CETP* provided evidence of lower genetically predicted effects on VLDL and LDL circulating concentrations. Notably, comparatively larger effects were identified on lipoprotein particle concentration and cholesterol concentrations within HDL subclasses with positive associations identified for these HDL-related traits. In contrast, marked heterogeneity was found in relation to triglycerides concentrations, with genetically predicted estimates suggesting an effect on higher very large and large HDL-C concentrations and on lower levels of medium and small HDL concentrations.

Genetically predicted triglyceride modifying targets (*APOC3*, *ANGPTL3*, *ANGPTL4*, and *LPL*) markedly lowered triglyceride concentrations across the spectrum of lipoprotein subclasses—this was in contrast to genetic instruments for *HMGCR*, *PCSK9*, *NPC1L1*, and *CETP* where effect estimates were weaker and tended to be on both sides of the null. For lipoprotein particle and cholesterol concentrations, lowering effects of triglyceride modifying targets were typically found for the VLDL-related traits, with an inflection point at IDL seen for *ANGTPL4*, *APOC3*, and *LPL* but not for *ANGPTL3*.

A comparison of these analyses repeated in the youngest and oldest subgroups using individual-level data from unrelated individuals within UKB (both *n =* 30,000) can be found in **S9 Fig**. Overall metabolic signatures did not appear to drastically differ between these strata defined by age, suggesting that treatment with statins was unlikely to lead to major perturbations in the effect estimates we present. While overall trends did not typically vary from those identified in the full sample, these findings suggest that analyses, which directly adjust for contingent factors within UKB, such as statin medications, are likely to introduce collider bias into their findings (as proposed previously [29]).

We also identified differing signatures between drug target classes for non-lipoprotein lipid–related traits. For instance, LDL lowering targets typically provided weak evidence of a genetically predicted effect on glycoprotein acetyls (GlycAs), a marker of inflammation (for instance, PCSK9: Beta = 0.01, 95 CI% = −0.06 to 0.08, *P* = 0.78). In contrast, all triglyceride lowering targets (i.e., *ANGPTL3*, *ANGPTL4*, *APOC3*, and *LPL*) provided strong evidence of a genetically predicted effect on lowering GlycA (for instance, LPL: Beta = −0.43, 95 CI% = −0.37 to −0.48, *P* = 9 × 10^−50^), as well as *CETP*. All drug target estimates on GlycA have been collated in **S15 Table**. Although GlycA is an adjunct of inflammation, we also provide genetically predicted effects of each target on C-reactive protein (CRP), measured using the biochemistry in the same participants with measures of NMR metabolites, given that it is a more widely and clinically used biomarker of inflammation (**S16 Table**). Similar directions of effect for targets on GlycA were found on CRP, although the only target to provide evidence of an effect robust to multiple comparisons was ANGPTL3 (Beta = −0.22, 95 CI% = −0.39 to −0.04, *P* = 0.02).

### Genetically predicted metabolic effects for drug targets in comparison to statin medication

**Fig 4:**
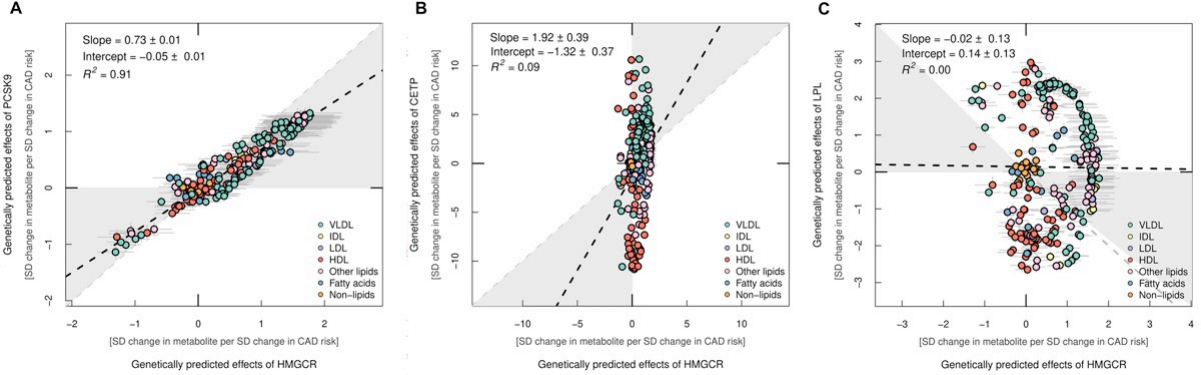
A comparison of distributions between genetically predicted drug target effects (A) PCSK9, (B) CETP, and (C) LPL on metabolic traits using NMR in the UKB study. In each figure estimates are compared with the results from the HMGCR score. Effect estimates were scaled in accordance with the corresponding effects of these genetically predicted drugs targets on risk of CAD, which is why axes vary between plots. Points are coloured based on subcategories of metabolic traits indicated in the figure legends. The data underlying this figure can be found in S7, S8, S10, and S14 Tables. CAD, coronary artery disease; CETP, cholesteryl ester transfer protein; HDL, high-density lipoprotein; IDL, intermediate density lipoprotein; LDL, low-density lipoprotein; NMR, nuclear magnetic resonance; SD, standard deviation; UKB, UK Biobank; VLDL, very low-density lipoprotein.

We systematically compared the genetically predicted effects of each drug target on all 249 metabolic traits with *HMGCR* acting as a proxy for statin therapy. For comparative purposes, estimates were scaled in accordance with their respective effect estimates on CAD as reported in **S2 Table**. In general, we identified strong evidence of concordance between the other LDL cholesterol lowering therapies (*PCKS9* and *NPC1L1*) with the *HMGCR* score (r^2^ = 0.91 and r^2^ = 0.79, respectively). **Fig 4A** illustrates the linear trend identified between genetically predicted effects of *PCSK9* and *HMGCR* on metabolic markers. **S10 Fig** contains the corresponding plot for *NPC1L1*.

In contrast, comparisons with the HDL cholesterol raising (*CETP*) (r^2^ = 0.09) (**Fig 4B**) and triglyceride lowering (*APOC3*, *ANGPTL3*, *ANGPTL4*, and *LPL*) (all r^2^ ≤ 0.02) targets provided weak evidence of concordance with the *HMGCR* score. **Fig 4C** visualises this general lack of concordance using the *LPL* and *HMGCR* score comparison as an exemplar. Broadly, both the *LPL* and *HMGCR* scores provided evidence of genetically predicted effects on higher levels of various triglyceride-rich VLDL-related traits (highlighted in green). However, conversely, the *LPL* score typically provided stronger evidence of an effect on HDL-related traits (highlighted in red), which was generally not the case for the *HMGCR* score. As expected, both scores provided weak evidence of an effect on non-lipid-related traits (highlighted in orange). All other figures generated from this analysis for the other targets in comparison to the *HMGCR* score can be found in **S11–S13 Figs**. There was also typically good concordance among metabolomic profiles derived from drug targets within the same lipoprotein lipid class, for instance, when comparing all pairwise combinations of triglyceride lowering drug targets (**S14–S19 Figs**). These findings were similar to those reported previously by a study conducted by Wang and colleagues [28], for example, with very strong concordance between *ANGPTL4* and *LPL* profiles identified (r^2^ = 0.96 by Wang and colleagues compared with r^2^ = 0.99 in this study). This is likely attributed to the large sample size harnessed in the study by Wang and colleagues (*n =* 61,240), which is also the case in this current study. *APOC3*, which was not evaluated by this previous study, likewise had a similar metabolomic profile as the other triglyceride lowering targets assessed (for instance, *LPL* and *APOC3* r^2^ = 0.94).

## Discussion

In this study, we explored the genetically predicted metabolic effects of modifying LDL, HDL, and triglycerides via drug targets that are either well established, recently licenced, and/or under development [30–33]. Our findings demonstrate that drug targets that principally act to modify LDL cholesterol (for instance, statins, PCSK9 inhibitors, and ezetimibe) have broadly similar effects on the blood metabolome. In contrast, effects of drugs designed to modify HDL cholesterol and triglycerides had very different effects on metabolic biomarkers, even when scaled to the same difference in risk of CAD. These findings provide a catalogue of genetically predicted pharmacological effects on the blood metabolome, which serves to illustrate the heterogeneity between different lipid-modifying therapies, highlighting the need for rich phenotyping of lipoprotein lipids in developing assays that gauge treatment response.

Our findings illustrate the tapestry of metabolic biomarker associations that are predicted to be downstream consequences of pharmacological modification of a therapeutic target. While these findings do not provide evidence of causation of these metabolic biomarkers, rather, they employ drug target MR as a means of characterising therapeutic effects on the metabolome [34,35]. Such diverse effects can then potentially be triangulated [36] to explore patterns of metabolomics where signatures are consistent with cardiovascular risk reduction. In-depth investigations into the independent causal role of specific metabolic traits at a granular level can then be explored using approaches such as multivariable MR [37]. For example, one might construct genetic instruments for biomarkers that are downstream consequences of HMGCR inhibition and conduct de novo multivariable MR analyses of these traits in order to identify the mediating mechanisms beyond apoB or LDL cholesterol. While previous studies, including those that we conducted, identified apoB as the fundamental driver of lipid-mediated CVD [7], a greater understanding of the causal components should facilitate new avenues of investigation and resultant pharmacological development. We identified important differences in the genetically predicted effects of some therapeutic targets on risks of CAD and T2D. HMGCR, NPC1L1 and PCSK9 all lowered risk of CAD, yet HMGCR, and to a lesser extent NPC1L1 and PCSK9, increased risk of T2D (as presented in Fig 2). In contrast, ANGPTL4 and LPL were genetically predicted to lower risks of both CAD and T2D. Comparisons of these therapeutic targets on detailed measurements using omics approaches such as those employed in this study may clarify the underlying aetiological mechanisms driving these differences and aid in the development of medicines that are protective for both vascular and metabolic diseases. For example, by exploring disease-specific effects of genetically instrumented drug targets and partitioning metabolic biomarkers according to such, it may be possible, through approaches such as multivariable MR, to identify in finer detail which metabolic biomarkers are causally implicated in CVD and metabolic disease. Additional approaches including “reverse-MR” [38,39], where genetic instruments for liability to disease are explored for their metabolomic signatures, may reveal biomarkers on the causal pathway to disease. Integration of these types of genetic epidemiological avenues of investigation are likely to resolve disease-specific roles of these metabolic biomarkers and, consequently, facilitate new therapeutic targets for clinical development. Additionally, our results provide granular insight into the genetically predicted effects on the plethora of circulating metabolic traits investigated in this study. For instance, the triglyceride lowering targets evaluated in this study typically provided strong evidence of an effect on GlycAs, a marker of inflammation, whereas in contrast, the LDL cholesterol lowering targets provided weak evidence of an effect on this circulating metabolic trait. This suggests that, although triglyceride lowering medications may not provide the same magnitude of effect towards lowering CAD risk as LDL lowering therapies, they may yield additional benefit towards reducing inflammation. Given that the role of inflammation in CVD is gaining traction as an orthogonal avenue of therapeutic potential, such effects of TG-modifying therapies on inflammation biomarkers offer potential therapeutic indications, which may have roles beyond CVD.

One of the striking findings is the general consistency of associations between drug targets and particle concentration and cholesterol concentration (likely owing to the high correlation between these phenotypic traits) and the divergence between particle and cholesterol concentration and triglycerides concentration. This was most notable when comparing drugs across their primary lipid indication—i.e., drugs that were developed on the basis of LDL cholesterol lowering tended to have modest associations with a general reduction in triglyceride concentrations across lipoprotein particles. In contrast, HDL cholesterol raising variants in *CETP* were identified to have effects on lower triglycerides in apoB containing lipoproteins, whereas for HDL particles, triglycerides were increased in very large and large HDL particles and reduced in medium and small HDL particles. Our *CETP* genetic score also had a genetically predicted effect on lower IDL and LDL lipoprotein particle and cholesterol concentrations, which has not been reported by previous MR evaluations of this target [19,40]. Possible explanations for this include the much larger number of genetic instruments leveraged in this study (*n =* 57), in comparison to previous studies that harnessed *n* ≤ 3 instruments, as well as performing analyses on a much larger sample size of individuals with NMR metabolites data in this work (*n* = 115,082).

For the drug targets where triglycerides metabolism was the primary lipid of pharmacological focus for development, triglycerides concentrations were lower across the lipoprotein particle spectrum. Since most of these drug targets demonstrated genetic evidence of CAD lowering, one might draw conclusions from such heterogeneity of triglycerides effects across these lipid-lowering therapies indicative that perhaps triglycerides were less important and that it was cholesterol or lipoprotein particle concentration (indexed, for instance, by apoB concentrations) that mediated these causal effects. However, previous multivariable MR analyses that included triglycerides, apoB, and LDL-C in the model demonstrated a direct effect of triglycerides consistent with a potential causal role of triglycerides in CAD [7,41] using the same dataset from the CARDIoGRAMplusC4D consortium as analysed in this study [42]. Thus, while our findings illustrate pronounced heterogeneity in cholesterol and triglyceride lipoprotein lipid concentrations arising from genetically predicted pharmacological inhibition of lipid modifying drug targets, drawing causal conclusions from such perturbations is nontrivial and requires MR of the individual phenotypes, as described previously.

The findings presented here have been made available by large-scale phenotyping using NMR-targeted metabolomics in UKB in combination with GWAS genotyping. Such data provide resolution of lipoprotein lipids at scale and enable genetic analyses of the type we present. The value of metabolomics may be to offer signatures of treatment response, which can then be used to guide pharmacological treatment. Such may be of utility from an early stage—for instance, during Phase I, II, and III clinical trials, where biomarkers are often used as a means of measuring treatment response across different concentrations of drugs [17], and postmarketing, when assessing interindividual response to treatment. Equally, our study has noteworthy limitations. For example, although previous studies have used similar criteria for instrument selection for the gene-based drug scores used in this study, we are unable to rule out genetic confounding as a potential source of bias in our analyses. Our analyses were also based on the European subset of the UKB study, and, therefore, evaluations in individuals of non-European ancestry would be valuable to investigate how representative our findings in diverse populations. Furthermore, we have used common genetic variants associated with lipoprotein lipid traits as a source of genetic instruments in this work. Future endeavours harnessing genetic effects on molecular traits (for instance, circulating proteins) or rare (and potentially highly penetrant) genetic variants may yield alternate strands of evidence to complement (or contradict) our results. In particular, these alternative approaches to genetic instrument selection for may identify a more powerful proxy for targets such as ANGPTL3 [43].

In summary, our study characterises the repertoire of genetically predicted lipid-modifying therapies on the blood metabolome. These findings demonstrate the widespread metabolic perturbance that arises from genetically evaluated modifications of therapeutic targets and heterogeneity between discrete classes of drugs, especially when their primary lipid trait differs. Such findings may be useful to illustrate the utility of drug target MR in gauging the predicted effects of drugs on omics traits to guide dose-ranging studies during clinical development and as a marker of treatment response.

## Materials and methods

### Instrument identification

Genetic instruments for each lipid-modifying drug target were selected by undertaking GWAS of lipoprotein lipid traits measured using a conventional biochemistry assay in UKB [44]. These included HDL cholesterol (field 30760), LDL cholesterol (field 30780), and triglycerides (field 30870). Details on genotyping quality control, phasing, and imputation in UKB have been described previously [45]. Briefly, GWAS were undertaken after excluding individuals with sex-mismatch (derived by comparing genetic sex and reported sex) or individuals with sex-chromosome aneuploidy were excluded from the analysis (*n =* 814). Next, a K-mean clustering algorithm was applied to remove UKB participants of non-European descent (based on K = 4) and also those with withdrawn consent leaving a maximum sample size of *n* = 463,005. Individuals who had measures of metabolic traits derived from a newly available NMR platform in UKB were also excluded from these GWAS (up to *n* = 121,727 participants) to avoid overlap with outcome samples (a potential source of bias in MR due to overfitting [20]). LDL cholesterol, HDL cholesterol, and triglycerides were normalised using inverse rank-normalisation such that their mean was 0 and their standard deviation was 1. We used the BOLT-LMM (linear mixed model) software with adjustment for age, sex, fasting status (i.e., the interval between consumption of food or drink and blood samples being taken), and a binary variable denoting the genotyping chip used in individuals (the UKBB Axiom array or the UK BiLEVE array) [46]. BOLT-LMM uses a linear mixed effect model to account for the population structure within UKB, which is why principal components were not included as covariates in the model. All analyses were conducted under UKB application #15825.

Final instrument selection for all 8 drug targets was based on results obtained from the GWAS of HDL cholesterol (to instrument *CETP*), LDL cholesterol (to instrument *PCSK9*, *HMGCR*, and *NPC1L1* [4]), and triglycerides (to instrument *APOC3*, *ANGPTL3*, *ANGPTL4*, and *LPL* [15,21]). A selection criteria of genetic variants with *P* < 1 × 10^−6^, which were located within a 100-kbs region either side of encoding genes, was applied to select instruments. This window size was selected to reduce the likelihood of including instruments proximal to other genetic targets, which may influence these lipoprotein lipid traits via alternate biological pathways (i.e., horizontal pleiotropy). We conducted linkage disequilibrium (LD) pruning for variants such that they had r^2^ < 0.1 using a reference panel of 503 European individuals enrolled in the 1,000 Genomes Project phase 3 (version 5) [47]. We additionally set out to identify genetic instruments for *PPARA* as a proxy for triglyceride modification through fibrates, although our GWAS only identified a single variant associated with triglyceride levels at this gene’s locus. We did not carry this target forward into downstream analyses given the challenges for genetic confounding of conducting gene-centric MR analyses with a single genetic instrument, particularly in gene dense regions where the function of each gene is not well understood, which may hinder inference [48].

### Genome-wide association studies of metabolic traits, coronary artery disease, and type 2 diabetes

We applied the same GWAS pipeline described above to all 249 metabolic traits measured by targeted high-throughput NMR metabolomics from Nightingale Health (biomarker quantification version 2020) in UKB. These analyses were conducted under UKB project #15825. Measures were taken using nonfasting EDTA plasma samples (aliquot) obtained from a random subsample of 121,584 UKB participants. Sample sizes on the 249 metabolic traits for GWAS after QC ranged between *n =* 110,051 to *n* = 115,082 UKB participants. A full summary of sample sizes can be found in **S3 Table**. Each metabolic trait was normalised to have a mean of 0 and standard deviation of 1 using inverse rank-normalisation as above allowing comparisons to be made between derived effect estimates. As before, all GWAS were adjusted for age, sex, fasting time, and genotyping chip.

Among these biomarkers were various lipoprotein lipids and their concentrations within 14 subclasses, fatty acids, ketone bodies, glycolysis metabolites, and amino acids (see **S3 Table**). Further details have been described previously [49]. Additionally, we conducted a GWAS of CRP measured using the biochemistry assay in UKB on the subset of participants with NMR metabolic traits. Ethical approval for this study was obtained from the Research Ethics Committee (REC; approval number: 11/NW/0382), and informed consent was collected from all participants enrolled in UKB.

Genome-wide summary-level estimates on CAD risk were obtained from a previously conducted GWAS from the CARDIoGRAMplusC4D consortium [42]. CAD cases in this consortium were defined as myocardial infarction, acute coronary syndrome, chronic stable angina, or coronary stenosis >50%. In total, there were 60,801 cases and 123,504 control assembled by CARDIoGRAMplusC4D across 48 studies, which did not include the UKB study. Genetic estimates on T2D were extracted from a previous GWAS from the DIAMANTE consortium consisting of 74,124 cases and 824,006 controls of European ancestry [50].

### Statistical analysis

#### Drug-target mendelian randomisation

Univariable MR analyses were firstly undertaken to estimate the genetically predicted effects of each therapeutic target on risk on CAD. Estimates were derived by applying the IVW method while accounting for the correlation between instruments using the same reference panel as above [22,23]. Further details on this approach have been described previously [22]. Briefly, we calculated the pairwise correlations between all variants included in genetic scores. These were then incorporated into the standard error terms of test statistics for the summary-level weighted generalised linear regression MR models.

This approach was then applied systematically to estimate the genetically predicted effects of each target on each of the 249 metabolic traits in turn. Analyses were conducted in a two-sample data setting to ensure that our sample of UKB participants from which instruments were identified (i.e., the non-NMR subset of UKB) did not overlap with individuals analysed in the GWAS of metabolic traits in UKB. To account for multiple testing, FDR corrections were applied for each drug target analysed as a heuristic to highlight the most noteworthy findings with strong statistical support based on current sample sizes, although all results are reported in the Supporting information (S1–S19 Figs, S1–S16 Tables). We repeated this analysis restricting our instrument selection criteria to a 50-kbs window around encoding genes for targets to assess the robustness of our findings to genetic confounding (i.e., variants influencing metabolic traits via neighbouring genes).

#### Comparison between different drug target effects across metabolome-wide traits

We initially compared effect estimates for a subset of lipid concentrations across drug targets using forest plots. More comprehensive comparisons on metabolome-wide results (i.e., on all 249 traits) were illustrated using scatter plots proposed previously to compare pairwise estimates between 2 targets with metabolic traits coloured based on their subcategories [28]. For comparative purposes, we scaled all metabolite estimates using a scaling factor based on each target’s corresponding genetically predicted effect on CAD risk. We used *HMGCR* estimates as our baseline comparison for each of the other 7 drug targets given the widespread adoption of statin therapy to treat individuals at elevated risk of CVD. Comparisons between *HMGCR* estimates and those for each of the other scores were evaluated using generated R^2^ values as applied previously [28]. R^2^ values are the coefficients of determination estimating the quotient of the variances of the fitted values and observed values of the dependent variable using a linear regression model. Here, it describes the linear fit for the estimates on metabolic traits between the 2 drug targets assessed.

As a sensitivity analysis, we used individual-level data from UKB to investigate whether the genetically predicted effects of drug targets on lipoprotein lipid concentrations varied among participant subgroups stratified by their age. As described previously [29], this approach permits the investigation of whether putative contingent factors in UKB may influence conclusions without directly conditioning of them. For example, in this study, we might anticipate that the influence of statin medications on metabolic markers may distort effect estimates. However, adjusting for this factor either as a covariate or by stratifying participants on it is likely to induce collider bias into analyses, which is recognised to potentially undermine causal inference [51]. As such, we partitioned the unrelated European sample from UKB into the youngest (range from 40 to 54 years) and oldest (range from 61 to 71 years) subgroups (both *n =* 30,000), where the number of reported participants taking statin medications was 5.6% and 27.7%, respectively. Instruments for drug targets were then constructed as genetic risk scores using individual-level data from UKB and analysed against each measure of lipoprotein lipid concentrations in turn using linear regression adjusted for age, sex, and the top 10 principal components.

All plots in this study were generated using the R package “ggplot2” [52]. MR analyses were conducted using the R package “MendelianRandomization” [53]. All analyses were undertaken using R (version 3.5.1).

## Supporting information

S1 Table Instrumental variables for drug targets in this study. (XLSX)

S2 Table Mendelian randomisation estimates on risk of coronary artery disease and type 2 diabetes. (XLSX)

S3 Table Mendelian randomisation estimates on lipoprotein lipid traits. (XLSX)

S4 Table Summary of circulating metabolic traits analysed in this study. (XLSX)

S5 Table Genome-wide significant results for circulating metabolic traits. (XLSX)

S6 Table Number of genome-wide significant hits for circulating metabolic traits. (XLSX)

S7 Table Mendelian randomisation estimates of the HMGCR drug score on circulating metabolic traits. (XLSX)

S8 Table Mendelian randomisation estimates of the PCSK9 drug score on circulating metabolic traits. (XLSX)

S9 Table Mendelian randomisation estimates of the NPC1L1 drug score on circulating metabolic traits. (XLSX)

S10 Table Mendelian randomisation estimates of the CETP drug score on circulating metabolic traits. (XLSX)

S11 Table Mendelian randomisation estimates of the ANGPTL3 drug score on circulating metabolic traits. (XLSX)

S12 Table Mendelian randomisation estimates of the ANGPTL4 drug score on circulating metabolic traits. (XLSX)

S13 Table Mendelian randomisation estimates of the APOC3 drug score on circulating metabolic traits. (XLSX)

S14 Table Mendelian randomisation estimates of the LPL drug score on circulating metabolic traits. (XLSX)

S15 Table Mendelian randomisation estimates of drug targets on glycoprotein acetyls. (XLSX)

S16 Table Mendelian randomisation estimates of drug targets on C-reactive protein levels. (XLSX)

S1 Fig Comparison of HMGCR metabolome-wide results using a flanking region of 50 kbs and 100 kbs around the encoding gene region. (PDF)

S2 Fig Comparison of PCSK9 metabolome-wide results using a flanking region of 50 kbs and 100 kbs around the encoding gene region. (PDF)

S3 Fig Comparison of NPC1L1 metabolome-wide results using a flanking region of 50 kbs and 100 kbs around the encoding gene region. (PDF)

S4 Fig Comparison of CETP metabolome-wide results using a flanking region of 50 kbs and 100 kbs around the encoding gene region. (PDF)

S5 Fig Comparison of ANGPTL3 metabolome-wide results using a flanking region of 50 kbs and 100 kbs around the encoding gene region. (PDF)

S6 Fig Comparison of ANGPTL4 metabolome-wide results using a flanking region of 50 kbs and 100 kbs around the encoding gene region. (PDF)

S7 Fig Comparison of APOC3 metabolome-wide results using a flanking region of 50 kbs and 100 kbs around the encoding gene region. (PDF)

S8 Fig Comparison of LPL metabolome-wide results using a flanking region of 50 kbs and 100 kbs around the encoding gene region. (PDF)

S9 Fig A comparison of drug target estimates on lipoprotein concentrations between analyses in the youngest and oldest 30,000 participants. (PNG)

S10 Fig Comparison of metabolome-wide results between HMGCR and NPC1L1 scores. (PDF)

S11 Fig Comparison of metabolome-wide results between HMGCR and ANGPTL3 scores. (PDF)

S12 Fig Comparison of metabolome-wide results between HMGCR and ANGPTL4 scores. (PDF)

S13 Fig Comparison of metabolome-wide results between HMGCR and APOC3 scores. (PDF)

S14 Fig Comparison of metabolome-wide results between ANGPTL3 and ANGPTL4 scores. (PDF)

S15 Fig Comparison of metabolome-wide results between ANGPTL3 and APOC3 scores. (PDF)

S16 Fig Comparison of metabolome-wide results between ANGPTL3 and LPL scores. (PDF)

S17 Fig Comparison of metabolome-wide results between ANGPTL4 and APOC3 scores. (PDF)

S18 Fig Comparison of metabolome-wide results between ANGPTL4 and LPL scores. (PDF)

S19 Fig Comparison of metabolome-wide results between APOC3 and LPL scores. (PDF)

## Abbreviations

ANGPLT3: angiopoietin-like protein 3
apoB: apolipoprotein B
CAD: coronary artery disease
CETP: cholesteryl ester transfer protein
CRP: C-reactive protein
CVD: cardiovascular disease
FDR: false discovery rate
GlycA: glycoprotein acetyl
GWAS: genome-wide association studies
HDL: high-density lipoprotein
IDL: intermediate density lipoprotein
IVW: inverse variance weighted
LD: linkage disequilibrium
LDL: low-density lipoprotein
MR: mendelian randomization
NMR: nuclear magnetic resonance
T2D: type 2 diabetes
UKB: UK Biobank
VLDL: very low-density lipoprotein
