# Supplementary figures and images for: Characterising metabolomic signatures of lipid-modifying therapies through drug target mendelian randomisation
Source: PLoS Biol. 2022 Feb 25;20(2):e3001547. doi: 10.1371/journal.pbio.3001547 (PMC8906647; doi:10.1371/journal.pbio.3001547)

Genetically predicted effects of HMGCR (50kbs)

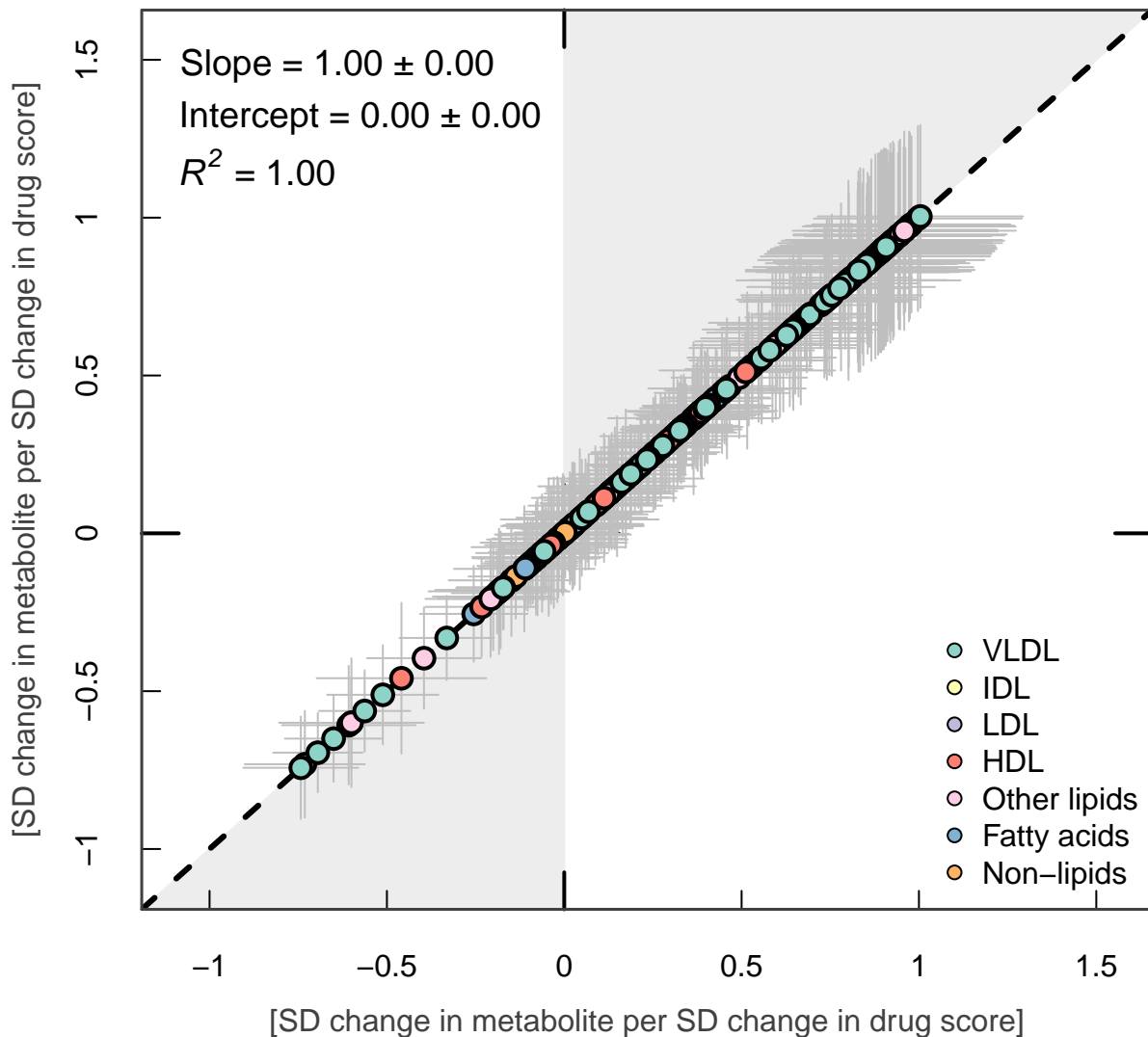

Genetically predicted effects of HMGCR (100kbs)

Supplement: S1 Fig — (PDF) [file pbio.3001547.s017.pdf]

Genetically predicted effects of PCSK9 (50kbs)

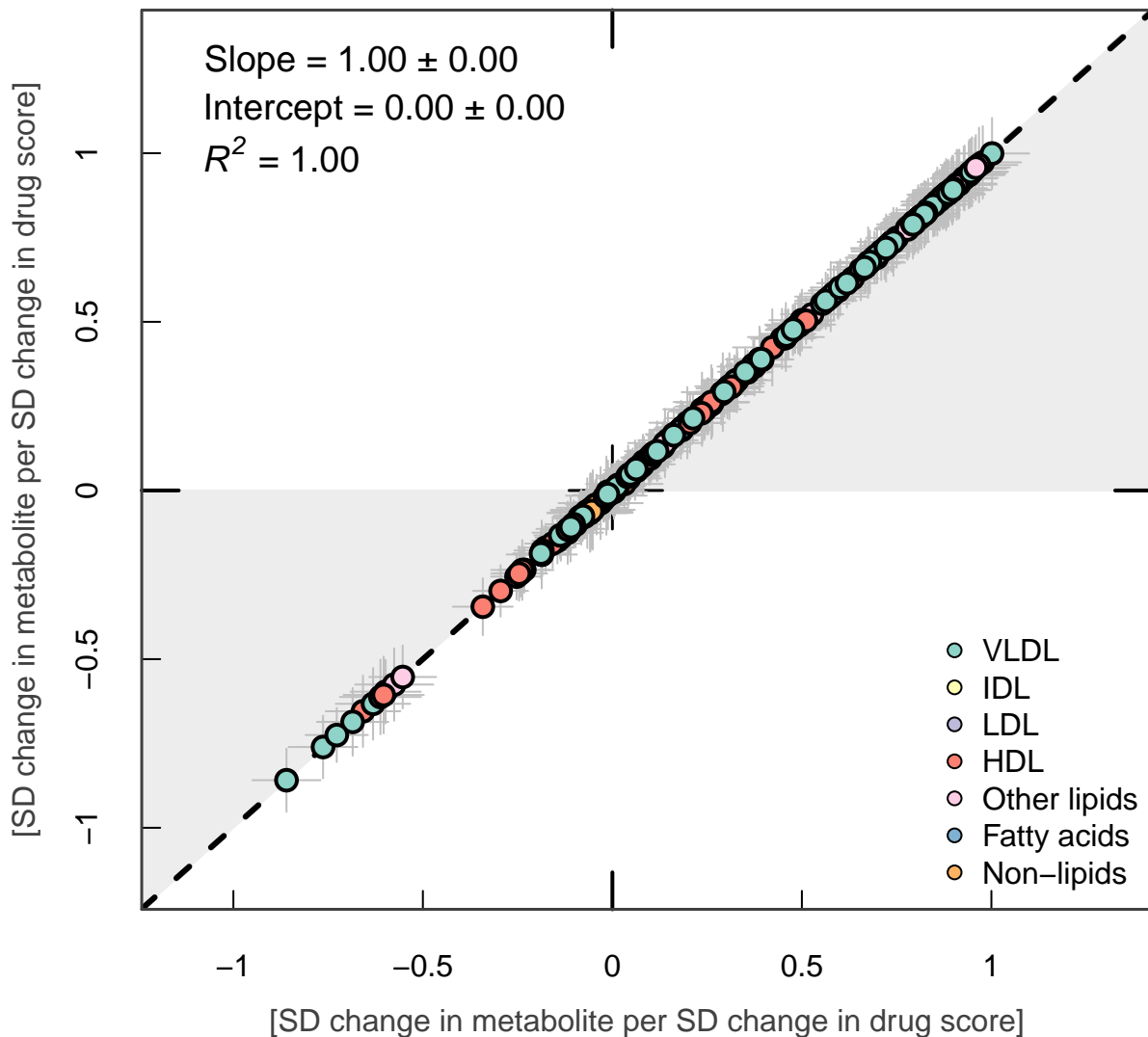

Genetically predicted effects of PCSK9 (100kbs)

Supplement: S2 Fig — (PDF) [file pbio.3001547.s018.pdf]

Genetically predicted effects of NPC1L1 (50kbs)

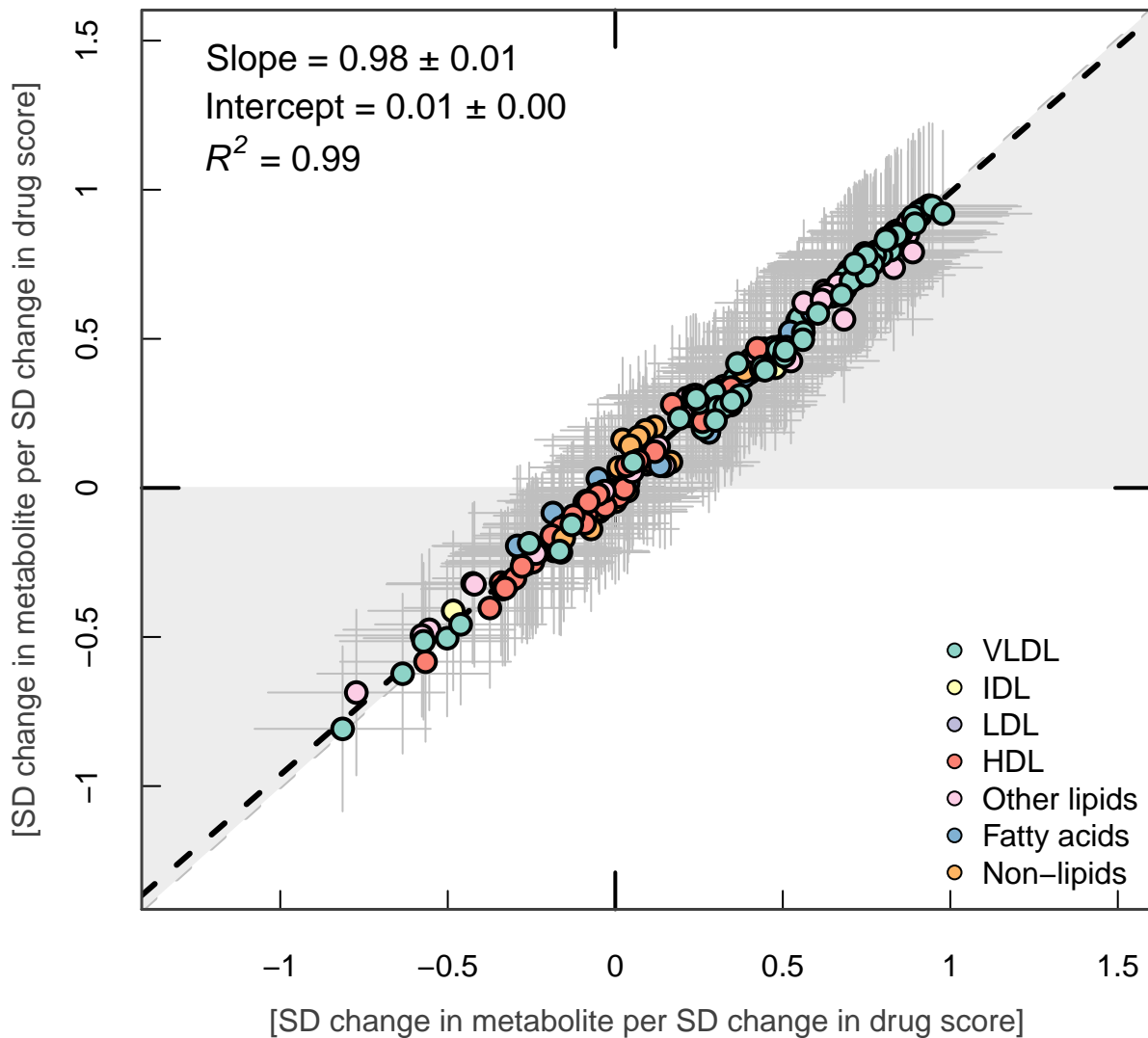

Genetically predicted effects of NPC1L1 (100kbs)

Supplement: S3 Fig — (PDF) [file pbio.3001547.s019.pdf]

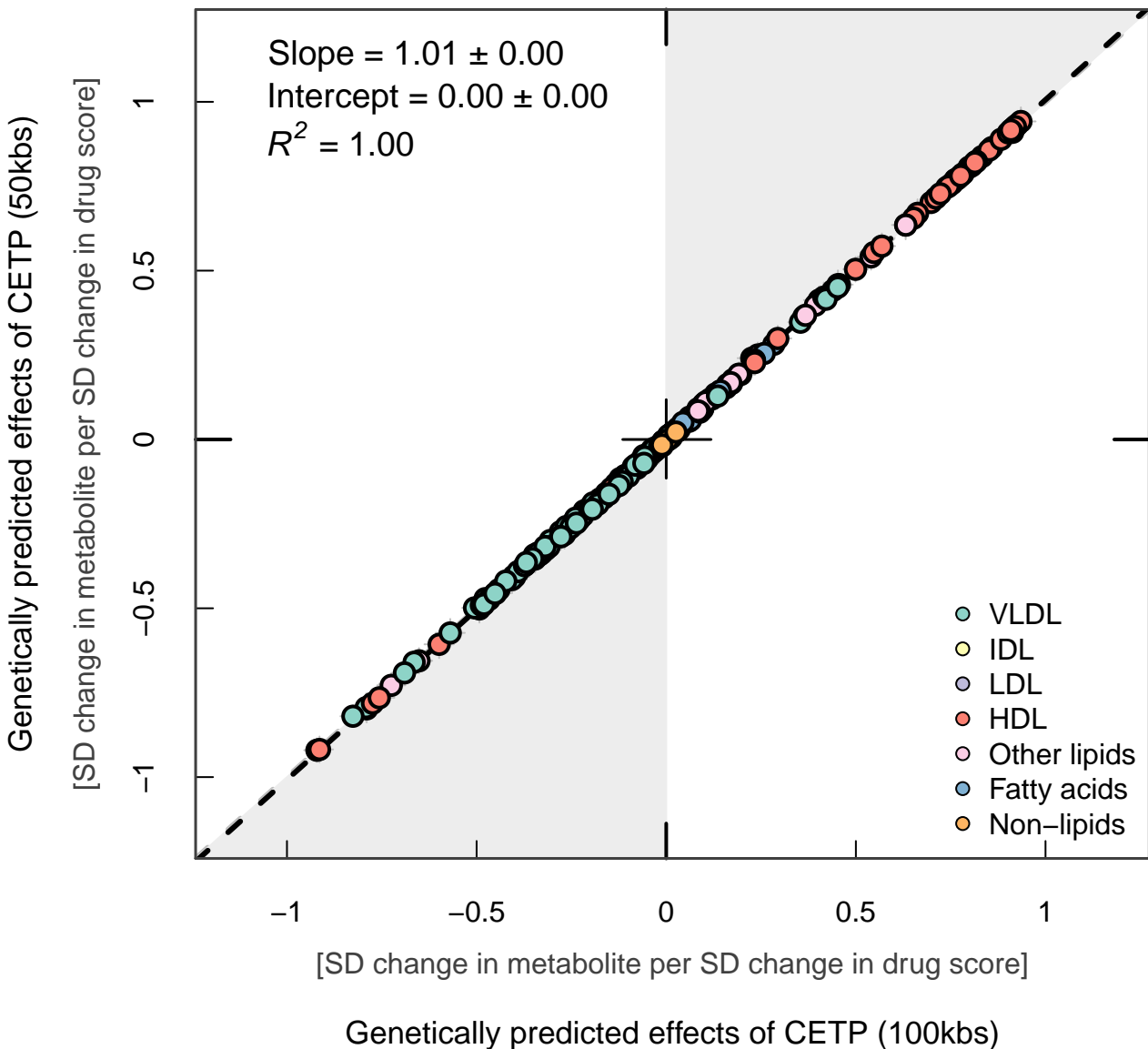

Supplement: S4 Fig — (PDF) [file pbio.3001547.s020.pdf]

Genetically predicted effects of ANGPTL3 (50kbs)

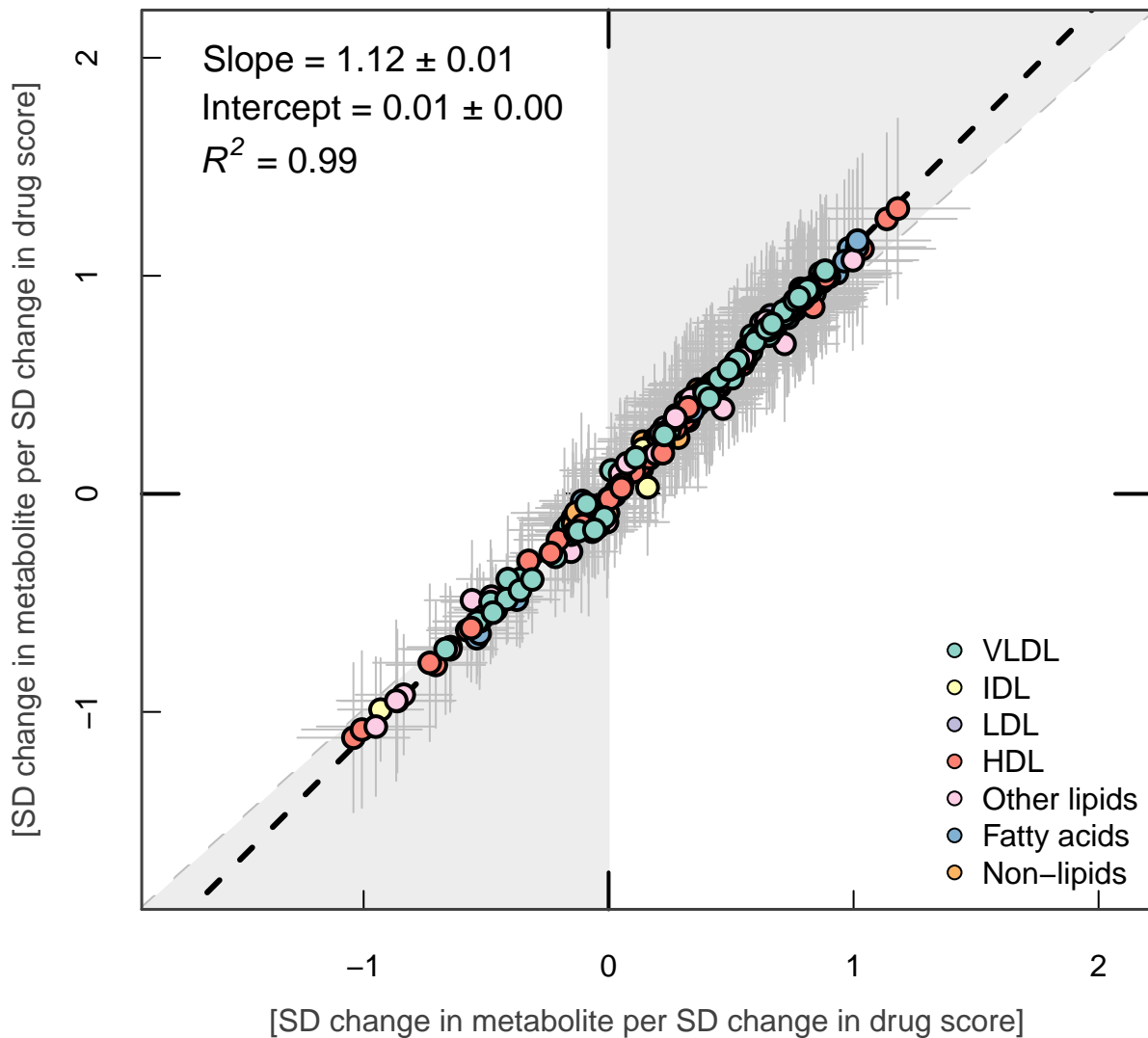

Supplement: S5 Fig — (PDF) [file pbio.3001547.s021.pdf]

Genetically predicted effects of APOC3 (50kbs)

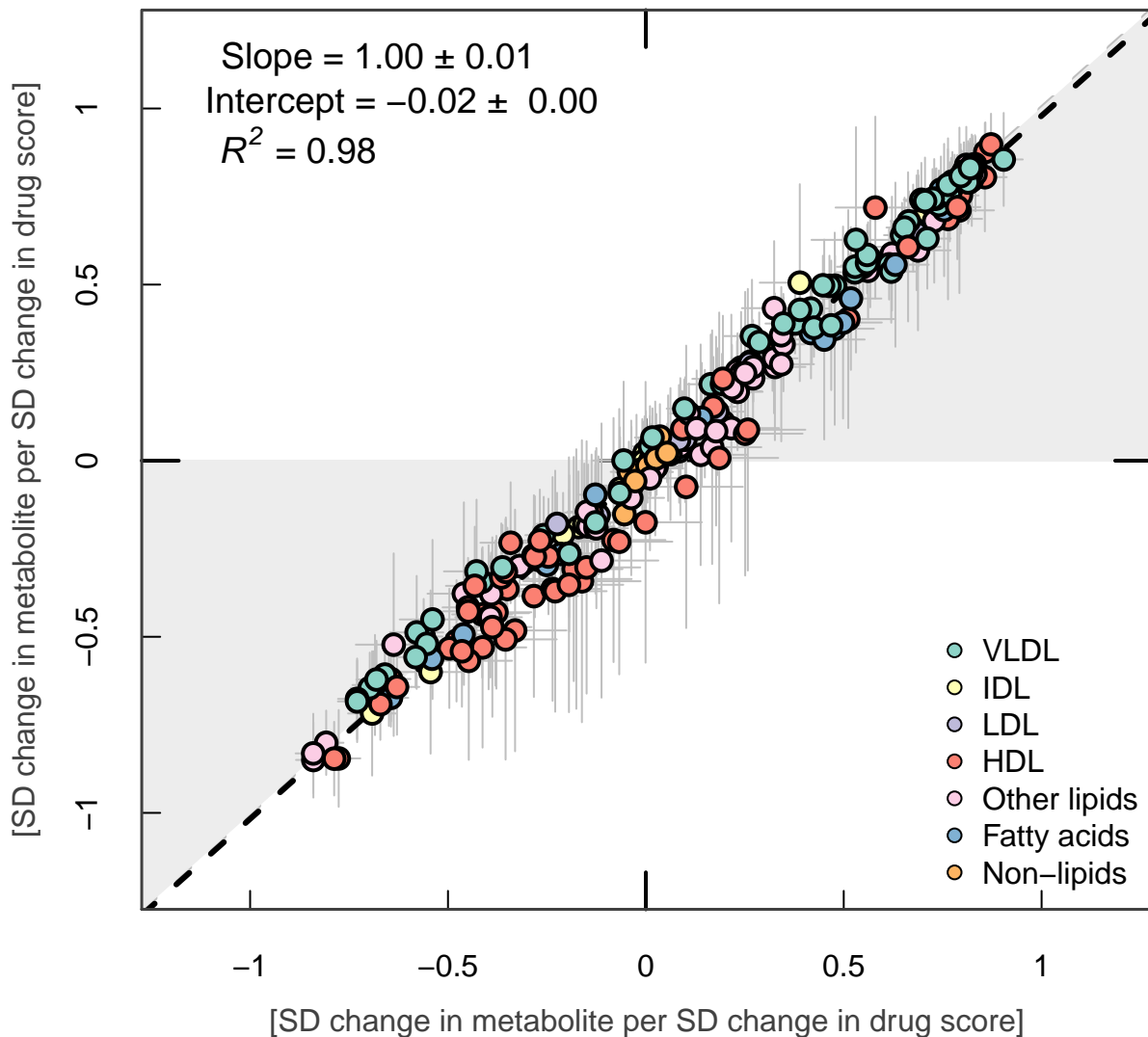

Genetically predicted effects of APOC3 (100kbs)

Supplement: S7 Fig — (PDF) [file pbio.3001547.s023.pdf]

Genetically predicted effects of LPL (50kbs)

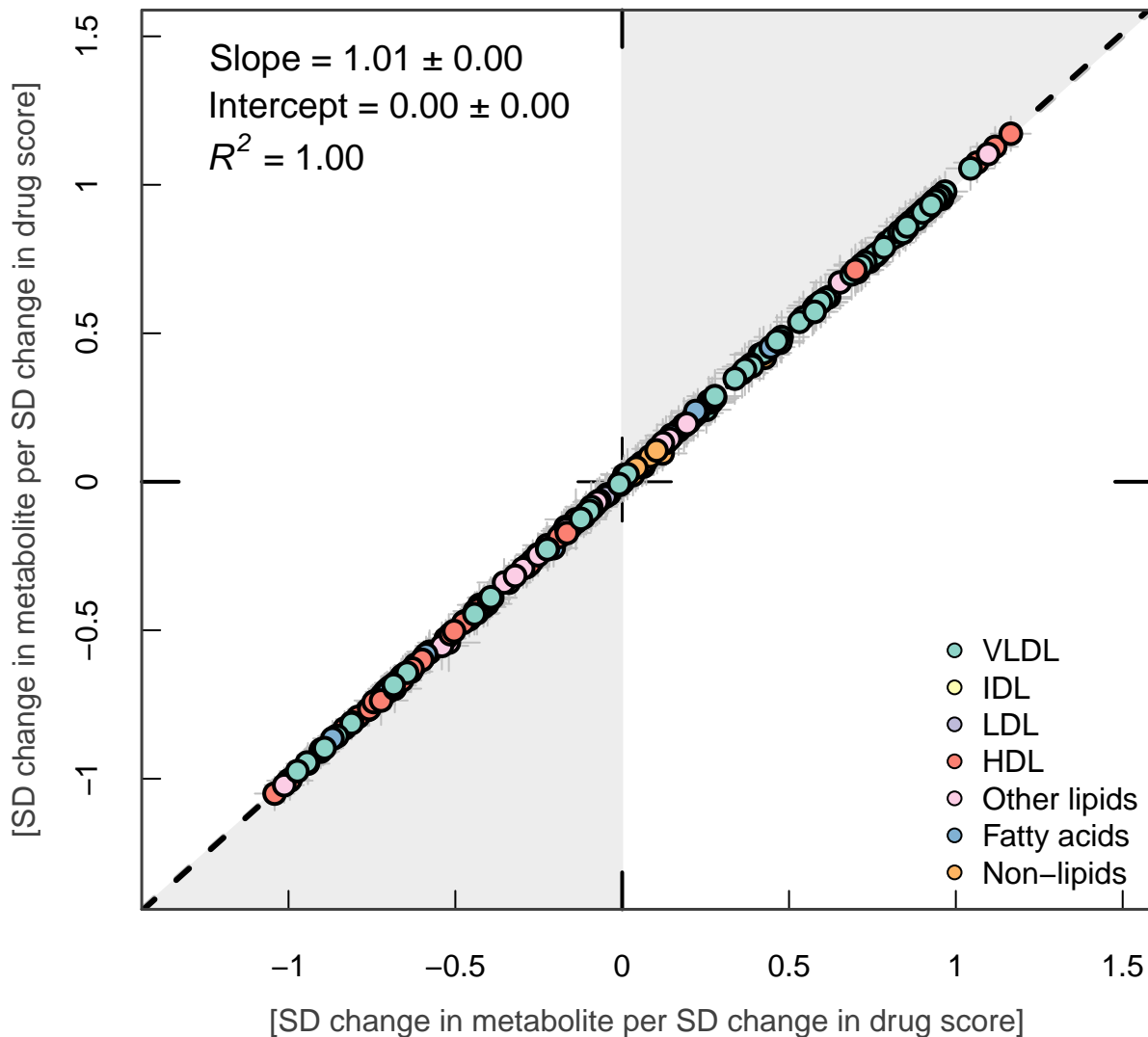

Genetically predicted effects of LPL (100kbs)

Supplement: S8 Fig — (PDF) [file pbio.3001547.s024.pdf]

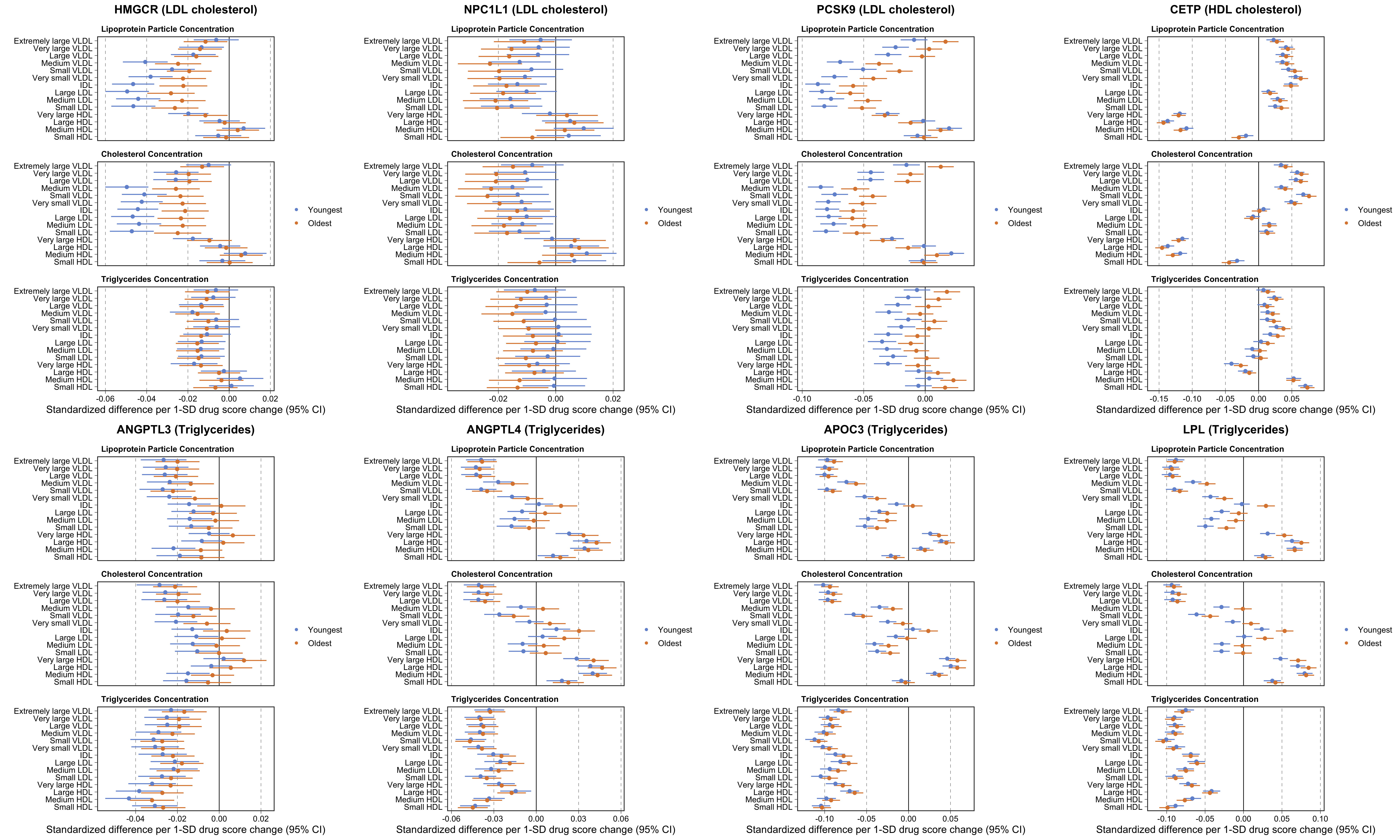

Supplement: S9 Fig — (PNG) [file pbio.3001547.s025.png]

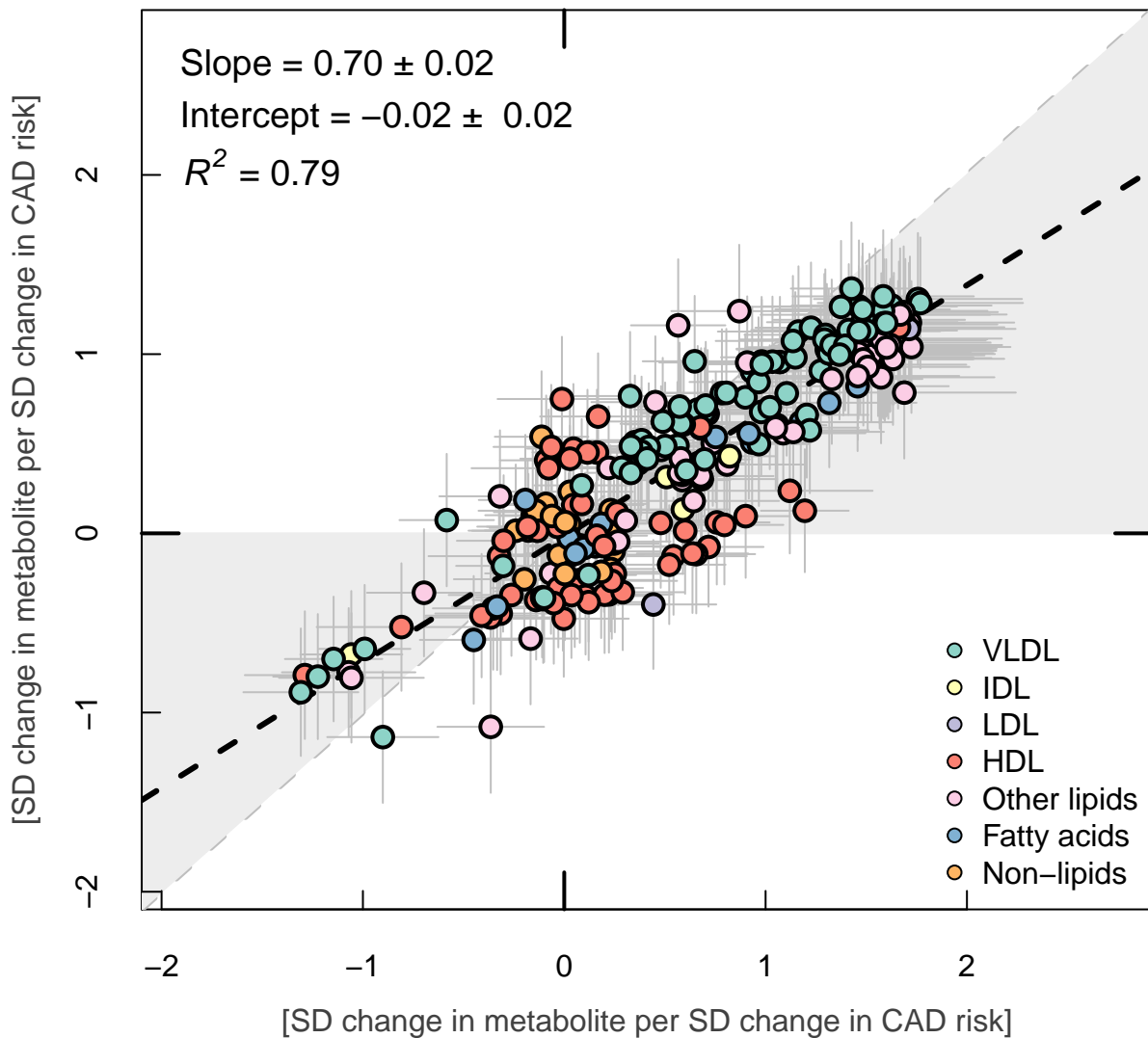

Supplement: S10 Fig — (PDF) [file pbio.3001547.s026.pdf]

Genetically predicted effects of ANGPTL3

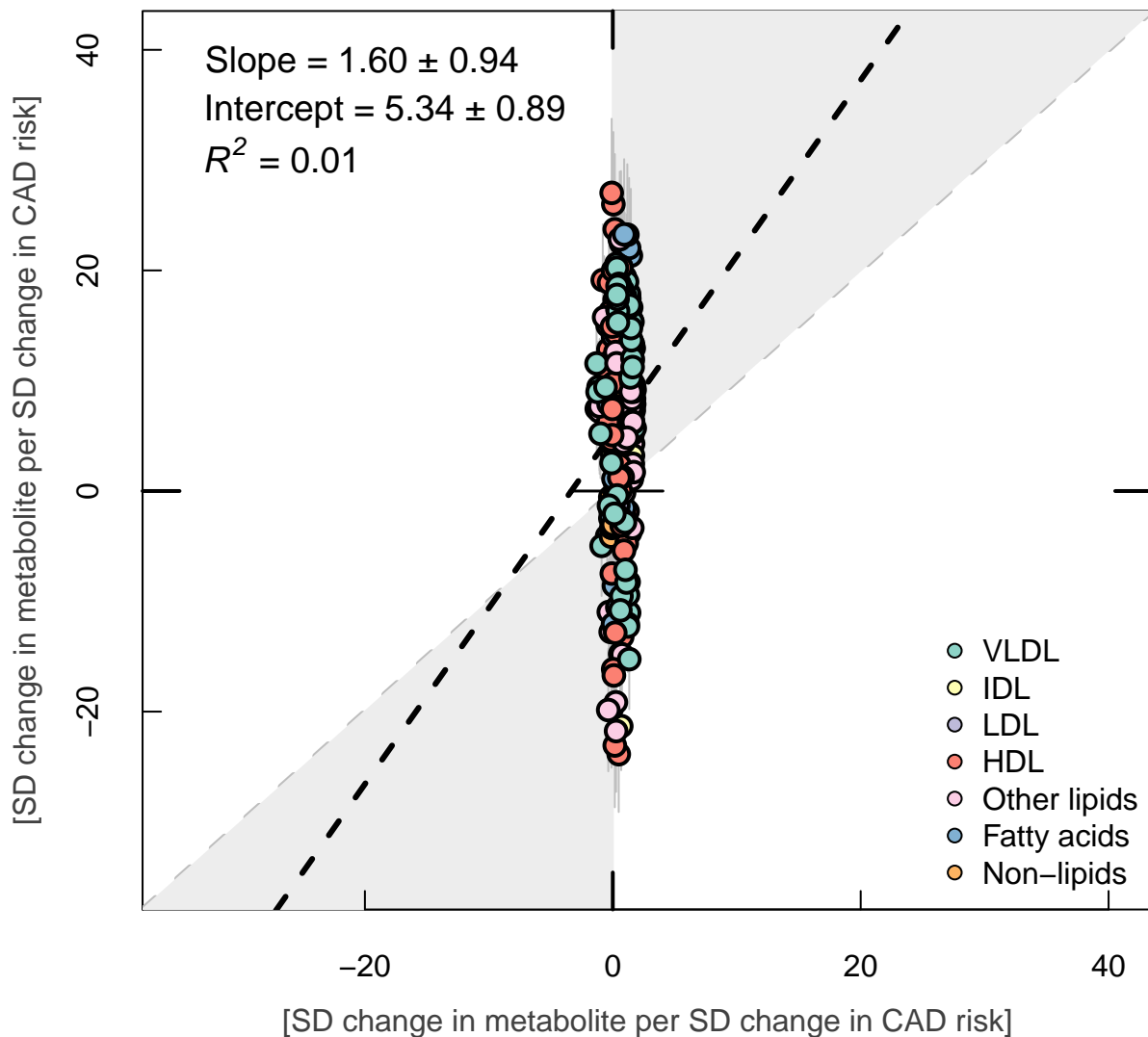

Supplement: S11 Fig — (PDF) [file pbio.3001547.s027.pdf]

Genetically predicted effects of ANGPTL4

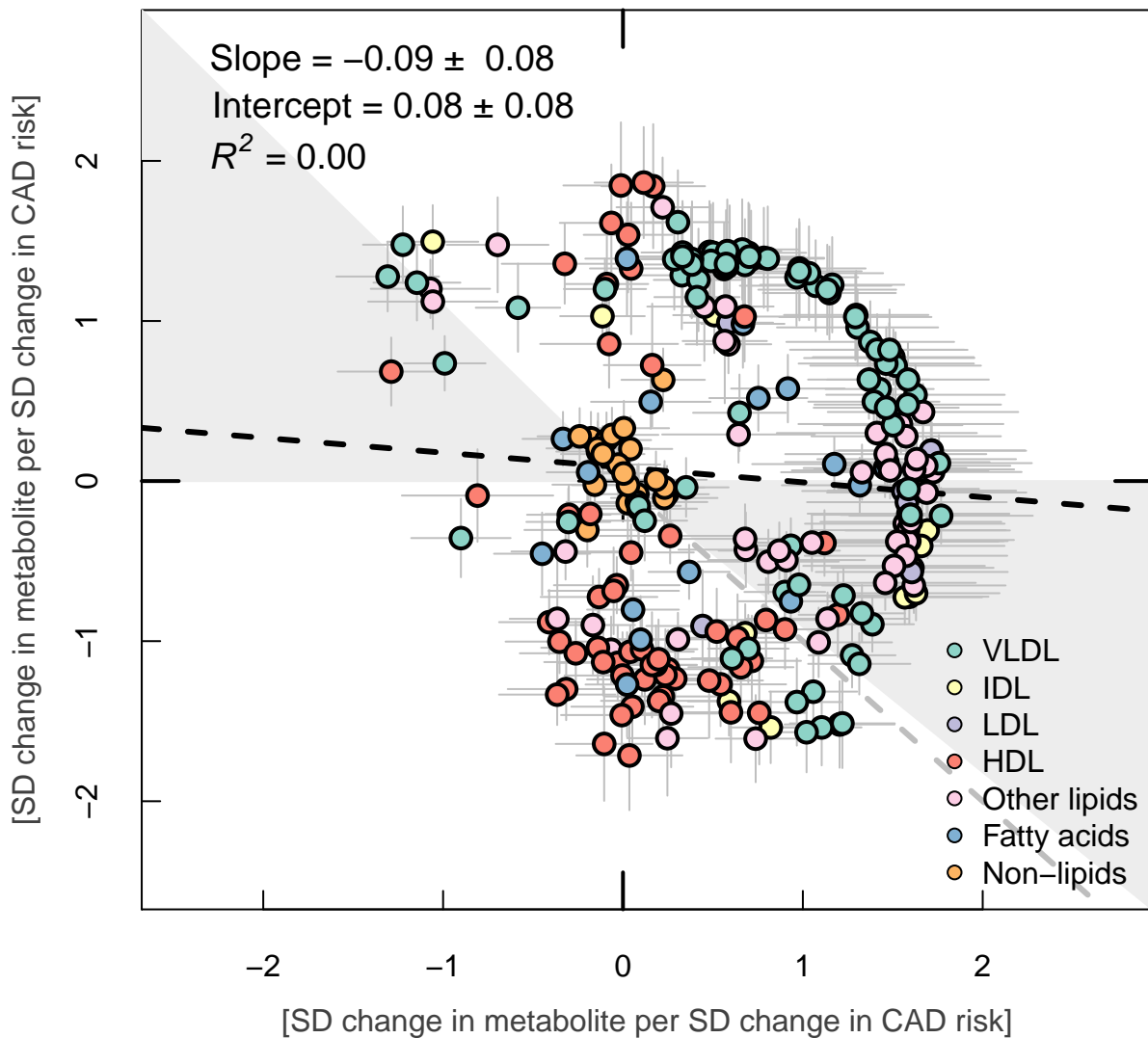

Genetically predicted effects of HMGCR

Supplement: S12 Fig — (PDF) [file pbio.3001547.s028.pdf]

Genetically predicted effects of APOC3

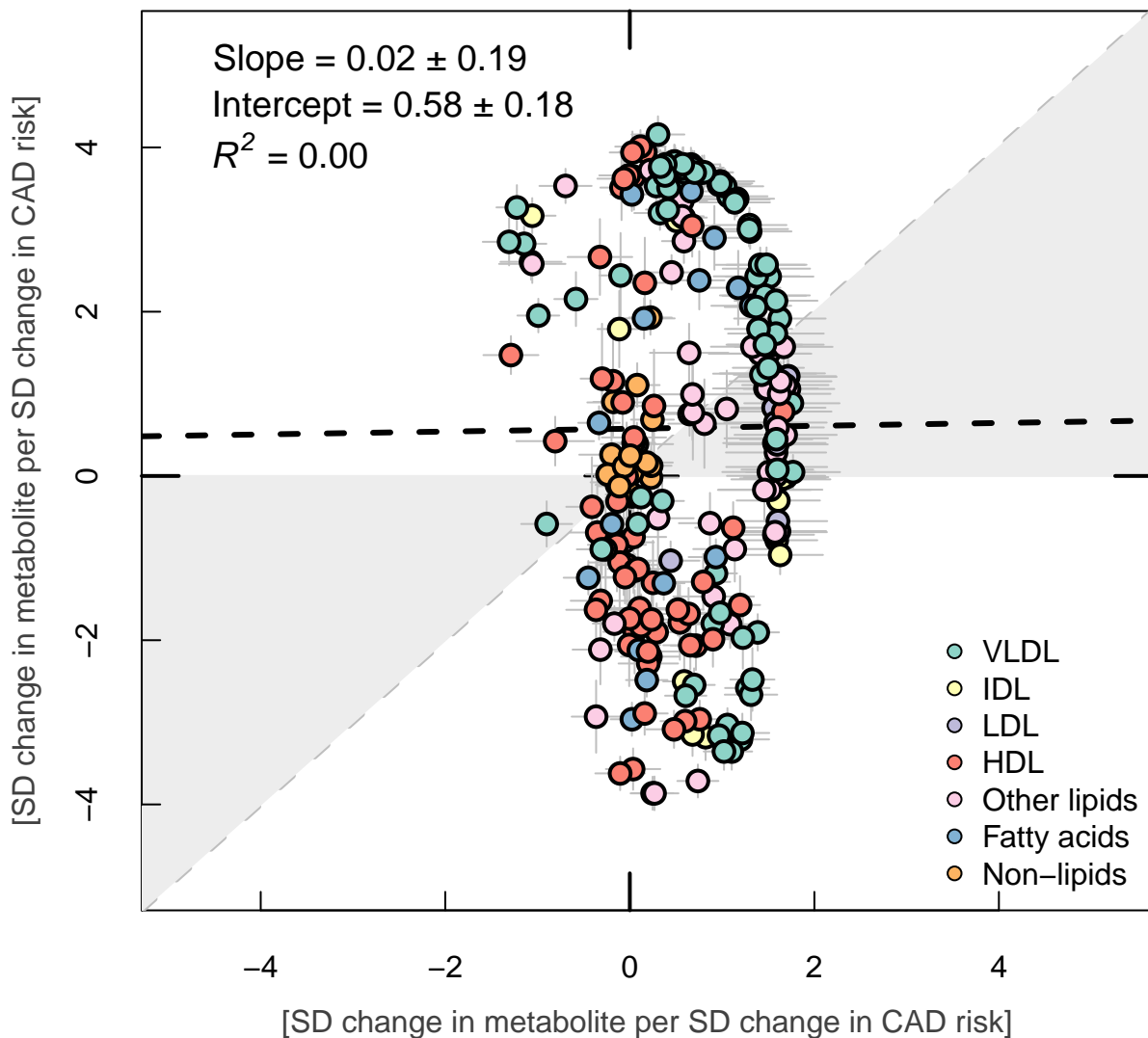

Supplement: S13 Fig — (PDF) [file pbio.3001547.s029.pdf]

Genetically predicted effects of ANGPTL4

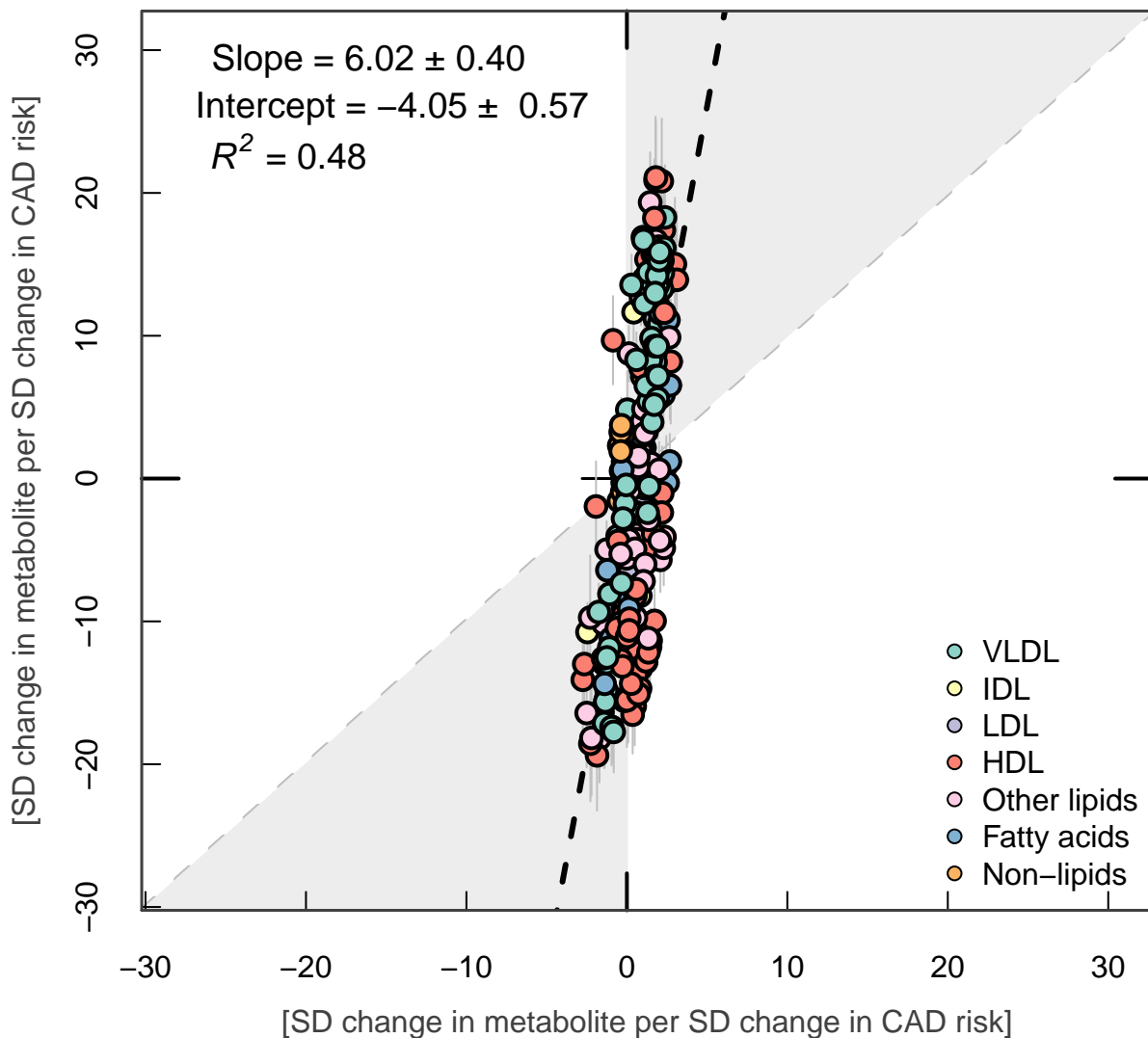

Genetically predicted effects of ANGPTL3

Supplement: S14 Fig — (PDF) [file pbio.3001547.s030.pdf]

Genetically predicted effects of APOC3

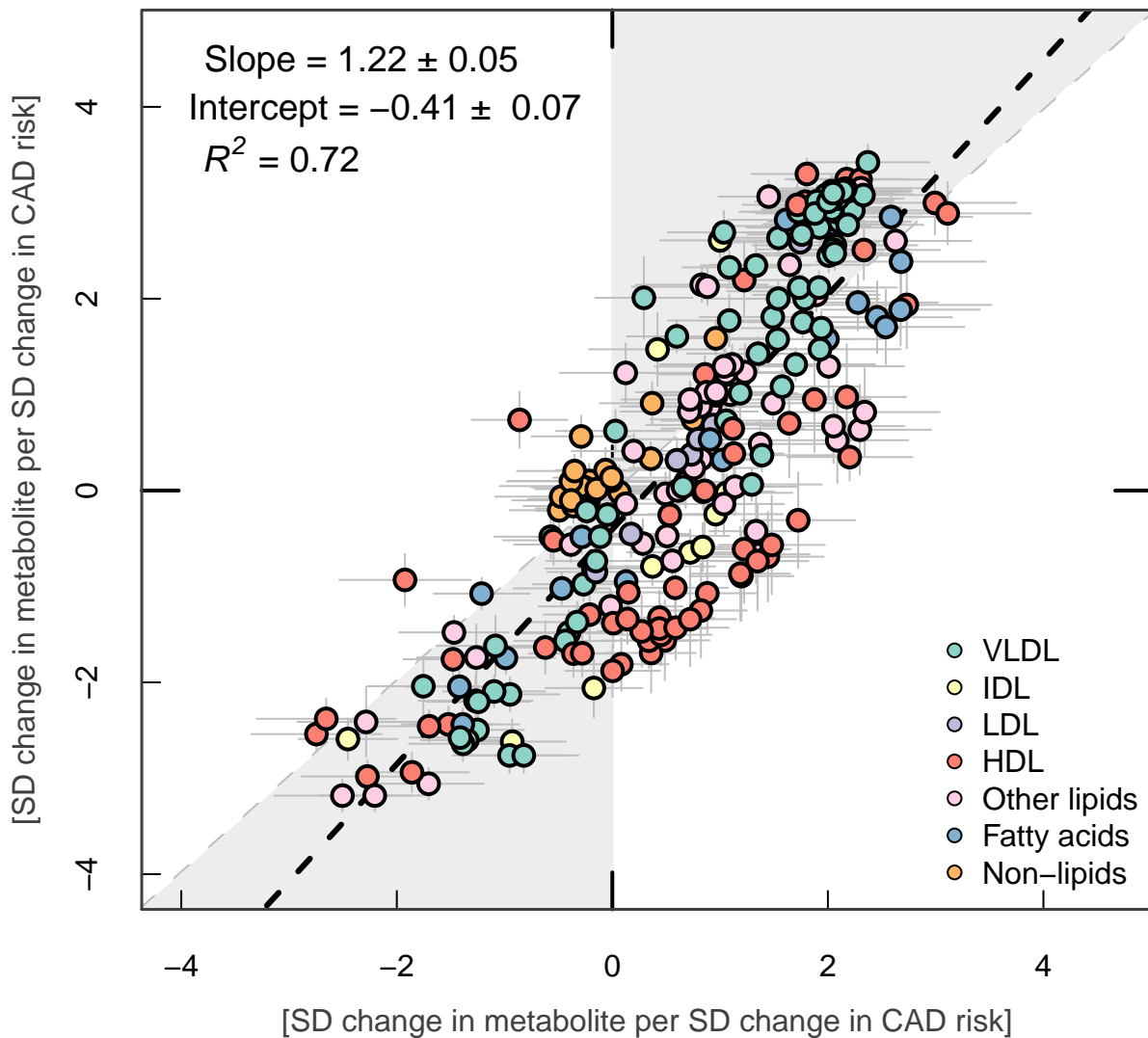

Supplement: S15 Fig — (PDF) [file pbio.3001547.s031.pdf]

Genetically predicted effects of LPL

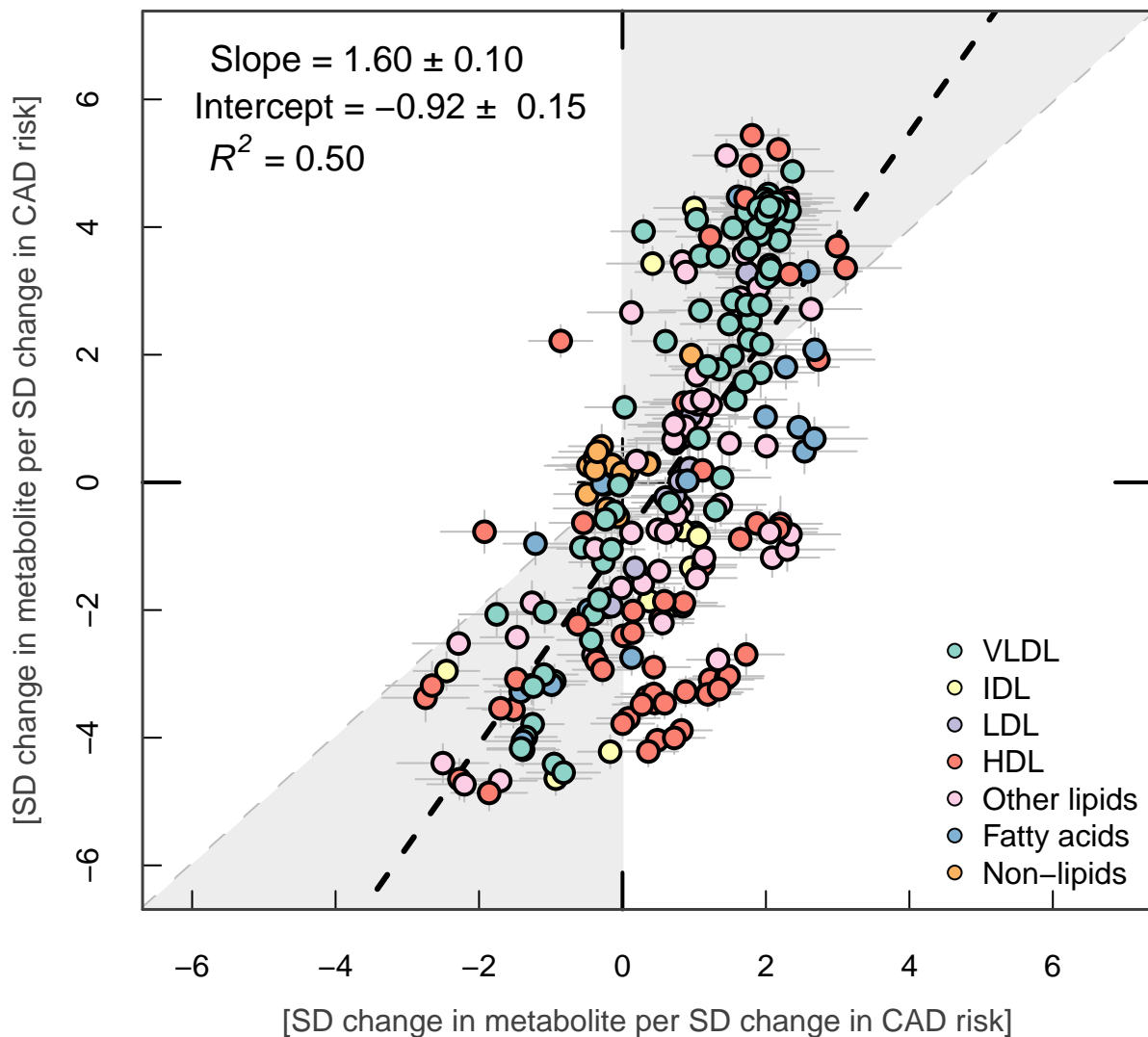

Genetically predicted effects of ANGPTL3

Supplement: S16 Fig — (PDF) [file pbio.3001547.s032.pdf]

Genetically predicted effects of APOC3

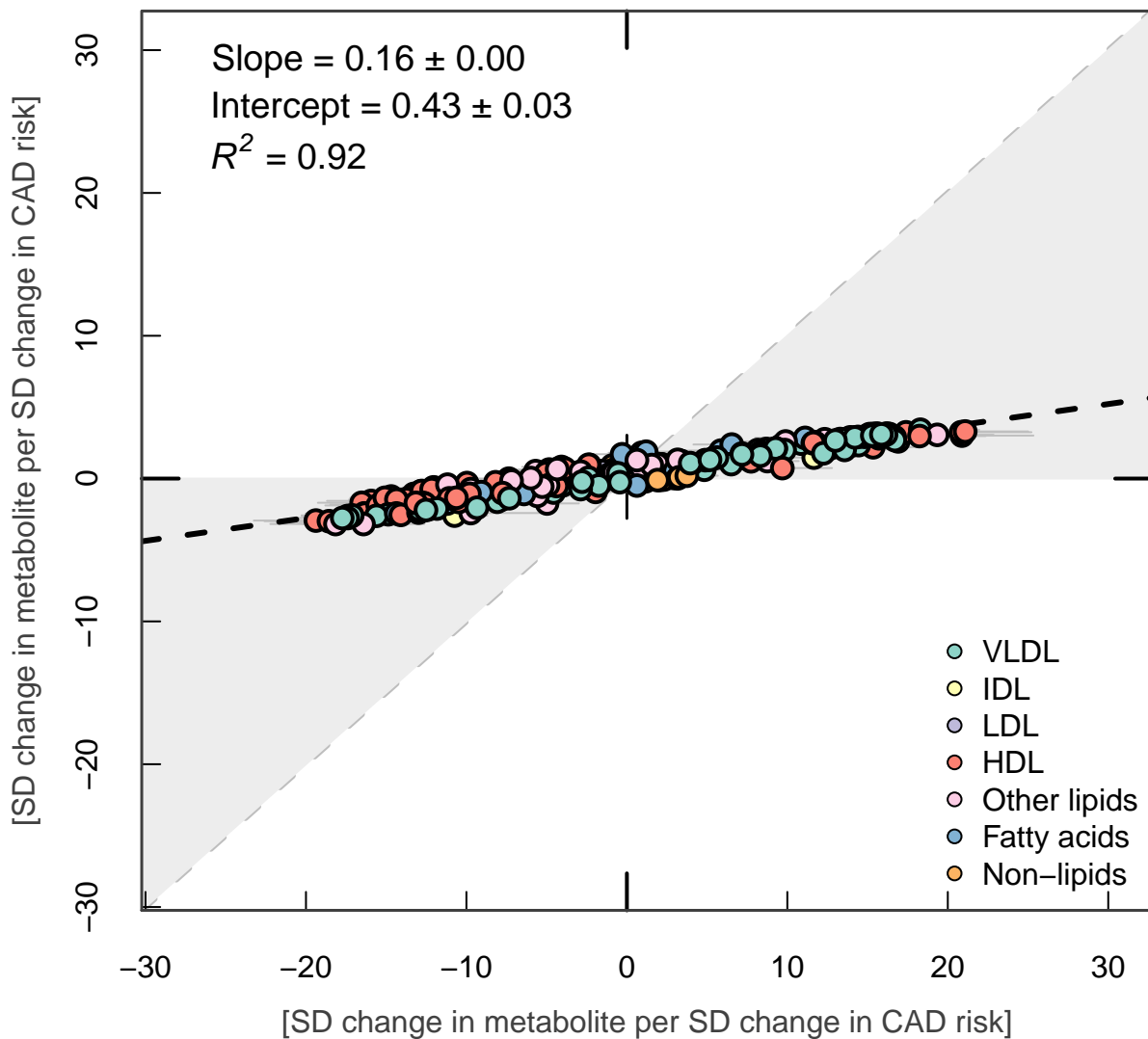

Genetically predicted effects of ANGPTL4

Supplement: S17 Fig — (PDF) [file pbio.3001547.s033.pdf]

Genetically predicted effects of LPL

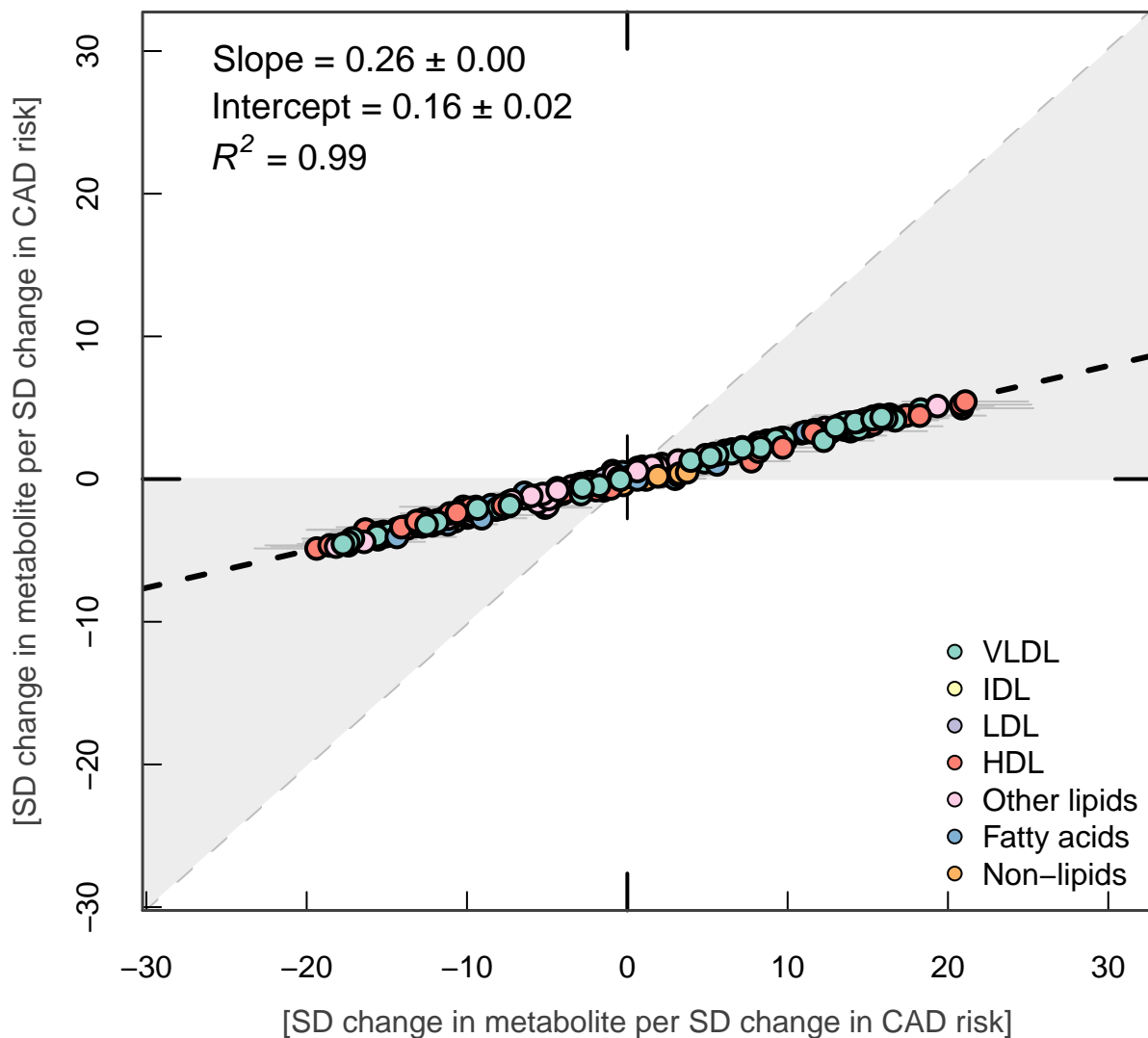

Supplement: S18 Fig — (PDF) [file pbio.3001547.s034.pdf]

Genetically predicted effects of LPL

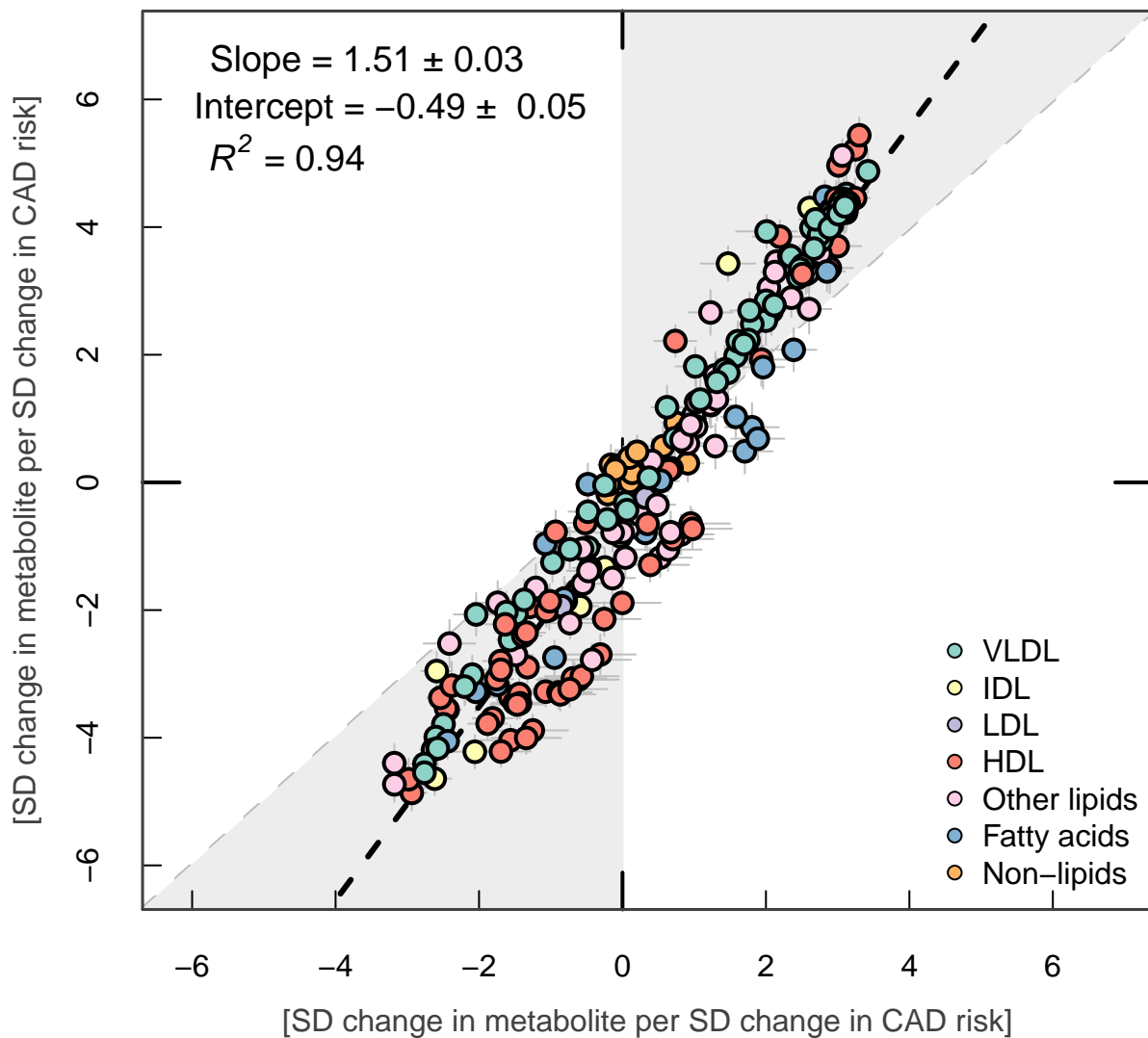

Supplement: S19 Fig — (PDF) [file pbio.3001547.s035.pdf]
