# Supplementary material for: Characterising metabolomic signatures of lipid-modifying therapies through drug target mendelian randomisation
Source: PLoS Biol. 2022 Feb 25;20(2):e3001547. doi: 10.1371/journal.pbio.3001547 (PMC8906647; doi:10.1371/journal.pbio.3001547)
Supplement: S6 Fig — (PDF) [file pbio.3001547.s022.pdf]

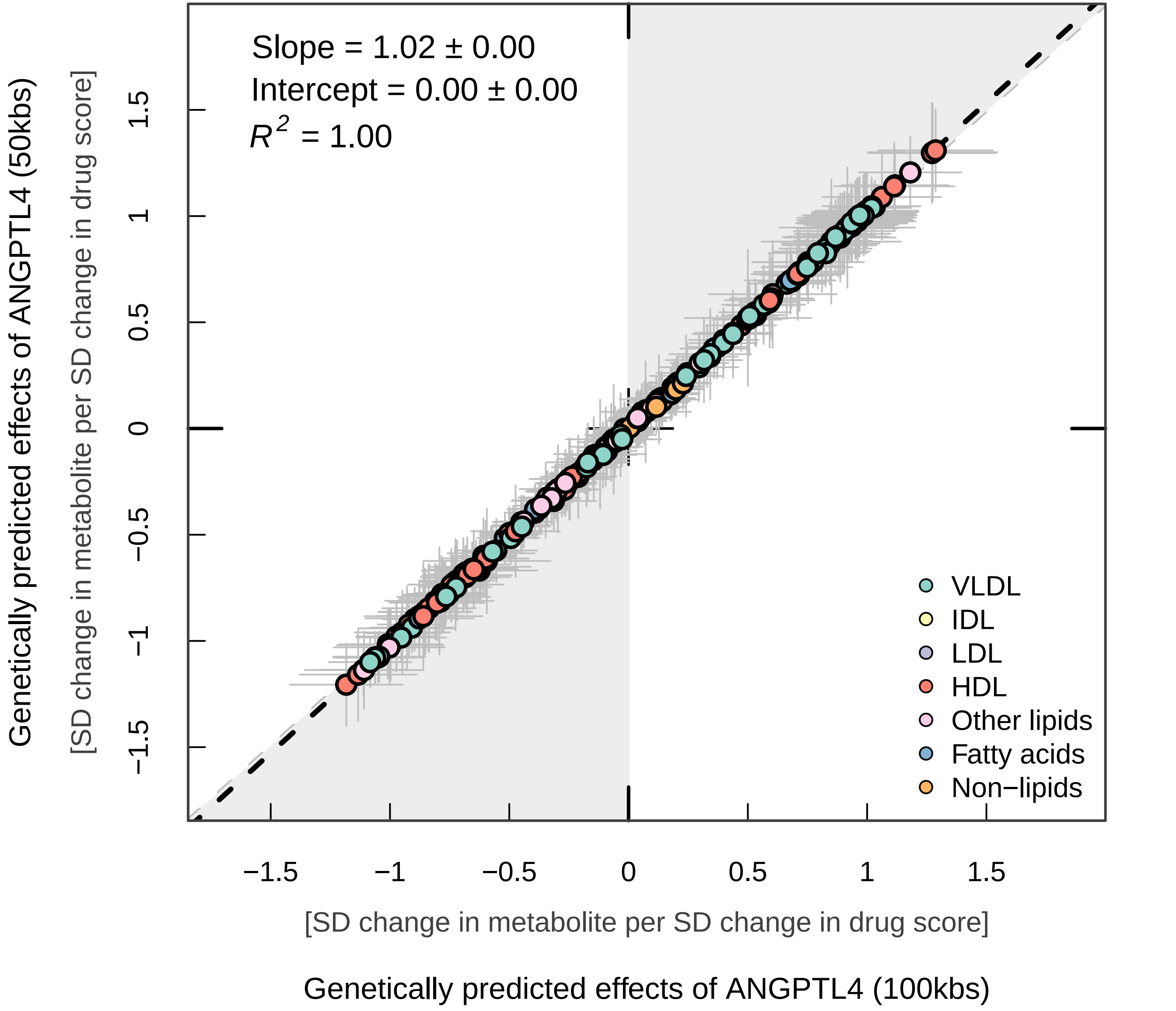

Slope = 1.02 ± 0.00
Intercept = 0.00 ± 0.00
R^2 = 1.00
VLDL
IDL
LDL
HDL
Other lipids
Fatty acids
Non−lipids
−1.5
−1
−0.5
0
0.5
1
1.5
Genetically predicted effects of ANGPTL4 (50kbs)
[SD change in metabolite per SD change in drug score]
Genetically predicted effects of ANGPTL4 (100kbs)
[SD change in metabolite per SD change in drug score]
